# Computational design and optimization of electro-physiological sensors

**DOI:** 10.1038/s41467-021-26442-1

**Published:** 2021-11-03

**Authors:** Aditya Shekhar Nittala, Andreas Karrenbauer, Arshad Khan, Tobias Kraus, Jürgen Steimle

**Affiliations:** 1 Human Computer Interaction Lab, Saarland University, Saarland Informatics Campus, Saarbrücken, 66123 Germany; 2 Max Planck Institute for Informatics, Saarland Informatics Campus, Saarbrücken, 66123 Germany; 3 INM - Leibniz Institute for New Materials, Saarbrücken, 66123 Germany

**Keywords:** Biomedical engineering, Computer science

## Abstract

Electro-physiological sensing devices are becoming increasingly common in diverse applications. However, designing such sensors in compact form factors and for high-quality signal acquisition is a challenging task even for experts, is typically done using heuristics, and requires extensive training. Our work proposes a computational approach for designing multi-modal electro-physiological sensors. By employing an optimization-based approach alongside an integrated predictive model for multiple modalities, compact sensors can be created which offer an optimal trade-off between high signal quality and small device size. The task is assisted by a graphical tool that allows to easily specify design preferences and to visually analyze the generated designs in real-time, enabling designer-in-the-loop optimization. Experimental results show high quantitative agreement between the prediction of the optimizer and experimentally collected physiological data. They demonstrate that generated designs can achieve an optimal balance between the size of the sensor and its signal acquisition capability, outperforming expert generated solutions.

## Abstract (web summary)

Though skin-conformable electro-physiological sensors are attractive for epidermal electronics, their optimal design remains a challenge. Here, the authors report a computational design approach for realizing multi-modal electro-physiological sensors that optimizes electrode layout design.

## Introduction

Measurement of biosignals through electrodes placed on the skin is common practice in specialized areas within medicine, health monitoring, and rehabilitation. Recent technical advances in highly ergonomic sensing devices now enable biosignal measurements to be accessible in more diverse contexts and to a larger audience. For example, advances in epidermal electronics have led to the development of skin-conformal devices that can monitor electrophysiological signals through skin-exposed electrodes^1–3^. Typically, electrodes are patterned on various ultra-thin substrates using functional materials^2,4–6^. Prior work has contributed epidermal sensors for capturing biosignals of various modalities, such as muscle movements using electromyogram (EMG)^2^, cardiac activity using electrocardiogram (ECG)^6^, or the electrical activity of the brain using electroencephalogram (EEG)^7^.

This new class of highly ergonomic devices promises to make electrophysiological sensing more widespread and opens up highly relevant new avenues in diverse fields, comprising wearable computing, augmented and virtual reality, entertainment computing, and human–machine interaction. However, designing sensor layouts for optimal acquisition of electrophysiological signals remains a hard problem, which currently limits a more widespread deployment of this technology. The exact placement of the sensing electrodes on the user’s body is critically important for acquiring high-quality signals^8^, as the quality of these signals often changes drastically even with small variations in the placement. Moreover, each biosignal poses unique requirements on valid body locations and electrode arrangements. These locations can further depend on an individual’s anatomical proportions, and hence differ across users^9^. This task is even more demanding if multiple biosignals are to be captured using one device. The current state-of-the-art is designing an electrode layout manually, using iterative trial-and-error by following a set of heuristic guidelines^9,10^. This manual approach is time-consuming and requires extensive domain expertise. Even with expert skills, electrode placements are known to be error prone^11^. Moreover, one of the key requirements for ergonomic wearability is a compact device form factor. At the same time, the device should be capable of acquiring signals with high quality. A good design solution should optimally trade-off between such conflicting design goals. Yet, manually finding such optimal trade-offs is typically not feasible due to the complex interplay of many parameters.

**Fig. 1:**
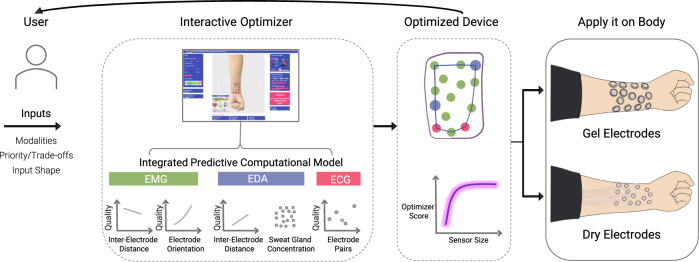
Overview of the concept of computational design and optimization of electro-physiological sensors. An integrated predictive model is presented, which encapsulates three biosignal modalities (EMG, EDA, and ECG). This model along with inputs from the user are fed to an optimizer which generates an optimized layout that optimally trades-off between desired device size and sensing quality. An interactive software tool assists the user in specifying desired properties and inspecting the generated design in real-time. The design can be further fine-tuned by an expert while interactively inspecting its quality, allowing for a “human-in-the-loop” optimization process. The optimized device can then be realized using commercial gel electrodes or through dry electrodes fabricated on a temporary tattoo.

We propose a computational design approach to tackle this problem (Fig. 1). It automates the design of electrode layouts for epidermal electrophysiological sensors that can sense biosignals of one or multiple modalities. It achieves two main goals: firstly, optimized sensor designs in compact form factors can be designed for supporting wearability and mobility, secondly, designs encapsulating electrodes which can measure multiple biosignal modalities can be rapidly realized taking into account multiple constraints. Based on the desired application, designs can be optimized not only for an individual user’s body but also for conflicting parameters such as signal quality and device footprint. An interactive design tool assists the user in easily specifying desired properties and aids in the rapid iterative design of multi-modal electrode layouts. To validate this approach, an optimization scheme has been designed and implemented for generating multi-modal electrode layouts, comprising three modalities: electromyography (EMG), electrodermal activity (EDA), and electrocardiogram (ECG). The optimizer has been conceived by formulating the electrode layout design process as a geometrical optimization problem.

Optimization techniques using physics-based models have been successfully employed for optimizing device designs in prior work, such as the design of actuators^12^, mechanical robots^13^, and optimized meta-materials^14^. The problem investigated here poses a new class of problems since biosignals depend on anatomical features of the human body. Therefore, an integrated predictive model has been devised that takes human anatomy into account to predict the sensing quality of multi-modal electrophysiological sensor designs. It comprises three biosignal modalities and can be operationalized for computational optimization. The main contribution here not only lies in applying geometrical optimization for tackling the problem of electrode placement but also in identifying, formalizing, and integrating the set of rules that are inherent to electrode placement for sensing multiple modalities. We show that an optimization approach can be employed for creating compact wearable devices that can measure multiple biosignal modalities.

The results presented here show that by using a computational design approach, multi-modal electrophysiological sensing layouts can be designed with considerably reduced device footprint while achieving high signal acquisition capability. The approach can rapidly identify optimal solutions for designs of complex combinations of electrodes for multiple modalities that comply with a desired device form factor—a task that so far was tedious and impractical even for experts. In the following, we use the placement of electrodes on the anterior side of the forearm as an example in order to demonstrate our approach and test its applicability by comparing it to conventionally obtained designs. First, we introduce an integrated predictive model for the three modalities EMG, EDA, and ECG that covers the anterior side of the forearm. The optimization problem is then formally introduced based on the model, and the algorithm is outlined. An optimizer was implemented with an interactive real-time graphical design tool that shields the user from lower-level details and exposes easy-to-use parameters for the design of sensors. Designs generated by the optimizer outperform the designs created by experts using conventional placement methods. Results from an experimental validation further show that a high quantitative agreement was found between experimentally collected physiological data from multiple subjects and the prediction of the optimizer. Finally, by unifying this optimization-based design strategy with multi-material inkjet printing, we demonstrate two application scenarios that provide a promising route towards a fully automated pipeline for the design and creation of complex multi-modal electrophysiological sensing devices.

## Results

Traditional manual placement of electro-physiological sensing electrodes relies on placing electrodes at specific locations, usually called keypoints, following a set of heuristic rules and placement guides presented in literature^9,15–18^. For multi-modal sensing, this typically results in either placing a dedicated device per modality at separate body locations or having large sensor sizes for sensing multiple modalities^19,20^. For improved wearability and mobility, we demonstrate a method based on computational optimization. The optimizer produces a single device that encapsulates electrodes that can measure multiple modalities and can be worn on the forearm. The forearm has been chosen as the location for this first study since it allows to capture multiple biosignals, supports ergonomic wearability^15,16,21–23^ and is one of the most promising areas for human-machine interaction^24–26^. However, this approach can also be applied to other modalities and body locations.

### Integrated predictive model

Computational design requires a formal model of electrode performance that can be operationalized for computational optimization. Furthermore, as the optimization approach searches for a globally optimal design of multi-modal sensors, this model needs to be compatible with multiple modalities. However, the current state of the art considers different modalities separately and uses incompatible metrics. For instance, the quality of an EMG signal is commonly measured in ARV (Average Rectified Value) or RMS (Root Mean Square) value of the signal, whereas EDA signals are measured through skin conductance levels denoted in MicroSiemens (*μ*S). This limitation is overcome with an integrated model that formalizes individual objective functions for each modality and defines cost functions for each, such that they can be combined in the overall objective function. The objective functions were formalized based on empirical findings reported from the literature for each modality. Here, we briefly discuss our approach for constructing the models. We first outline our approach for EMG and then very briefly introduce the main concepts underlying the models for EDA and ECG. The details of the entire model are described in the “Methods” section.

Electromyography (EMG) measures the MUAP (Motor Unit Action Potential) as an electrical potential between a ground electrode and sensing electrodes. The Surface-EMG (sEMG) measurement is a typical non-invasive method to capture MUAP by placing electrodes on the surface of the skin. For a given muscle, the EMG signal is captured by a pair of electrodes with respect to a reference electrode. The signal quality depends on a number of factors such as the electrode size, its placement with respect to the muscle line and the distance between electrodes. From an optimization perspective, the overall optimizer score for a given muscle is normalized in the range [0,1], with 0 denoting the best and 1 denoting the worst sensing quality. Our current implementation supports five muscles on the anterior side of the forearm: Flexor Carpi Radialis (FCR), Brachioradialis (BR), Palmaris Longus (PL), Pronator Quadratus (PQ), and Flexor Carpi Ulnaris (FCU).

Input parameters of the model are a set of forearm measurements *F* = {*f*_1_, *f*_2_, *f*_3_, *f*_4_} (Supplementary Fig. 1), a weight *w*_1_ determining the priority for EMG in the overall objective function, and optionally the desired shape of the sensor *S*. Using *F*, the five muscle lines can be reconstructed based on the guides from prior work^9,17,27^. To ensure good signal acquisition, the model incorporates electrodes that have a surface area of 50 mm^2^^9,10,28^.

Following successful checks for electrode distances from the muscle lines and their non-presence within Innervation Zones (IZ) (which are unsuitable locations for placing electrodes)^17^, a normalized score is calculated based on the electrode orientation with respect to muscle and the inter-electrode distance. For a given muscle *i*, the overall energy function is calculated as follows:

1 $${O}_{1i}=\left\{\begin{array}{ll}\alpha \cdot \omega ({\theta }_{i})+(1-\alpha )\cdot \nu (| {\overrightarrow{e}}_{i}| )&\,{{\mbox{if}}}\,\ {e}_{i}^{\prime},{e}_{i}^{{\prime\prime} }\,\notin\, {R}_{i}\\ {\hskip -110pt}1&\kern-0.5pc {{\mbox{otherwise}}}\,\end{array}\right.$$

where *ω*(*θ*_*i*_) and $\nu (| {\overrightarrow{e}}_{i}| )$ represent cost functions for the electrode orientation *θ* and inter-electrode distance ${\overrightarrow{e}}_{i}={e}_{i}^{\prime}-{e}_{i}^{{\prime\prime} }$, respectively, *α* and 1 − *α* are the respective priorities, and *R*_*i*_ corresponds the innervation zones which are not suitable locations for the electrode placement. In the current model, equal priorities (*α* = 0.5) are given for both these factors. The full details of the model are described in detail in the Methods section.

For *n* selected muscles, the overall EMG score is then calculated as the product of the EMG weight assigned and the average of the scores of all selected muscles, which can be formulated as:

2 $${O}_{1}(F,{E}_{1},{w}_{1},S)={w}_{1}\cdot \frac{1}{m}\mathop{\sum }\limits_{i=1}^{m}{O}_{1i}$$

where *F* = {*f*_1_, *f*_2_, *f*_3_, *f*_4_} is the set of forearm measurements, *E*_1_ is the set of electrodes for EMG, *w*_1_ is the weight determining the priority for EMG in the overall objective function, and *S* is the optional input shape.

Electrodermal activity (EDA) measures the changes in electrical conductance of the skin and has been used as an indicator for detecting emotional responses^29^. The EDA response is influenced by the sweat gland activity, which is directly proportional to the number of sweat glands (higher the number of sweat glands, higher the skin conductance levels). Hence the EDA model predicts the number of sweat glands covered between the electrodes, which determines the quality of the EDA response. The details of the model are presented in the “Methods” section.

Electrocardiogram (ECG) measures the electrical activity that occurs during a cardiac cycle. Clinically, the measurements are obtained by placing 12 electrodes near the chest^18^. More recently, three-electrode ECG configurations on the forearm have been designed to support ambulatory and wearable devices^16,21,23,30^. Our model is derived based on the mapping of ECG signals at various locations on the forearm as described in prior work^16^. The details of the model can be found in the “Methods” section.

To ensure that devices with small form factors are created, an additional weight *w*_4_ for small area is incorporated into the model. This small area weight *w*_4_ determines the priority given to the size of the device. The details of this model can be found in the “Methods” section.

### Computational optimization

The predictive model provides the basis of a computational design tool that can generate a sensor design that packs a set of electrodes for measuring one or more electrophysiological signals. Each pair of electrodes have a specific functionality; for instance, two electrodes placed on the muscle measure electric potential generated from muscle movements, while another electrode pair measures electrodermal activity, etc. The spatial configuration of the electrodes affects the quality of the biosignals which are to be acquired. The aim of the optimizer is to find a globally optimal solution that provides a good trade-off between signal quality and the overall size of the sensor.

The model allows for specifying which biosignals the sensor should be able to capture. Any combination of EMG, EDA, and ECG can be selected. The choice of EMG involving specifying the set of individual muscles for sensing. The designer can further specify weights for prioritizing or de-prioritizing biosignals in global optimization. A higher priority implies that this biosignal is given more weight, increasing the likelihood the corresponding electrodes are placed such that high-quality sensing is ensured. Similarly, the designer can specify a weight indicating how aggressively the optimizer seeks to create small form factor solutions. Furthermore, if desired, the designer can specify the exact outline and position on the body that any valid design must not exceed. In this case of constrained optimization, the optimizer searches for optimal solutions only within the given input shape *S,* which is a closed polygon.

The optimization problem was formulated as follows. Given a set of forearm measurements *F* = {*f*_1_, *f*_2_, *f*_3_, *f*_4_} (Supplementary Fig. 1), an input shape *S*, and a set of weights *W* = {*w*_1_, *w*_2_, *w*_3_, *w*_4_} which represent the priorities for EMG, EDA, ECG and Small Area respectively (*w*_1_ + *w*_2_ + *w*_3_ = 1), an electrode set *E* = {*e*_1_, *e*_2_, . . , *e*_*n*_} is generated. The overall global objective function of the electrode set *E* is:

3 $$O(F,W,S)=\mathop{\sum }\limits_{k=1}^{4}{w}_{k}\cdot {O}_{k}$$

which is minimized over all non-overlapping placements of the electrodes in *E* within the input shape *S*, where *O*_1_, *O*_2_, *O*_3_, *O*_4_ are the objective functions for EMG, EDA, ECG, and small area, respectively.

Considering the challenge of dealing with a large search space, a large number of potential solutions are possible. Monte-Carlo approaches are well suited for these kinds of problems, where sampling the entire solution space is not feasible. Here, efficient sampling of new configurations with an objective function that can be evaluated quickly can result in well-optimized solutions. Hence, simulated annealing (SA)^31^ was used for implementing the optimization scheme. It is a probabilistic technique for approximating the global optimum of a given energy function. The annealing procedure starts with a random initial layout that is generated within the shape *S*. After every iteration, a neighboring layout is generated by picking a random electrode and translating it with a vector $\overrightarrow{v}$. The new solution is accepted if it either lowers the objective or raises it based on a randomized probability function which is given as follows:

*c* ← *rand*(0, 1)

**if** $$c\le {e}^{\frac{-{{\Delta }}O}{T}}$$ **then**

*accept* *solution*

**else**

*reject* *solution*

**end** **if** where Δ*O* = *O*(*t*) − *O*(*t* − 1) is the difference in the objective function at successive annealing temperatures, and *T* is the annealing temperature.

In addition to the weight-based optimization approach in which the user provides relative priorities through weights, an additional optimization scheme has been incorporated. In this lower-bound based-optimization, the user specifies a required reference signal value for each of the modalities. The optimizer then strives for higher quality scores than the respective lower bounds for each modality. The user can control the hardness of the lower bound constraints by a parameter, i.e., violations are penalized as described in the “Methods” section. In this scheme, the relative weights of the modalities can be disabled such that only the area weight is used for calculating the objective function. Hence, each modality receives equal priority for achieving a quality above the corresponding lower bound. However, the user can also activate lower bounds and modality weights at the same time in a hybrid scheme.

### Conception of an interactive optimizer with a software tool

**Fig. 2:**
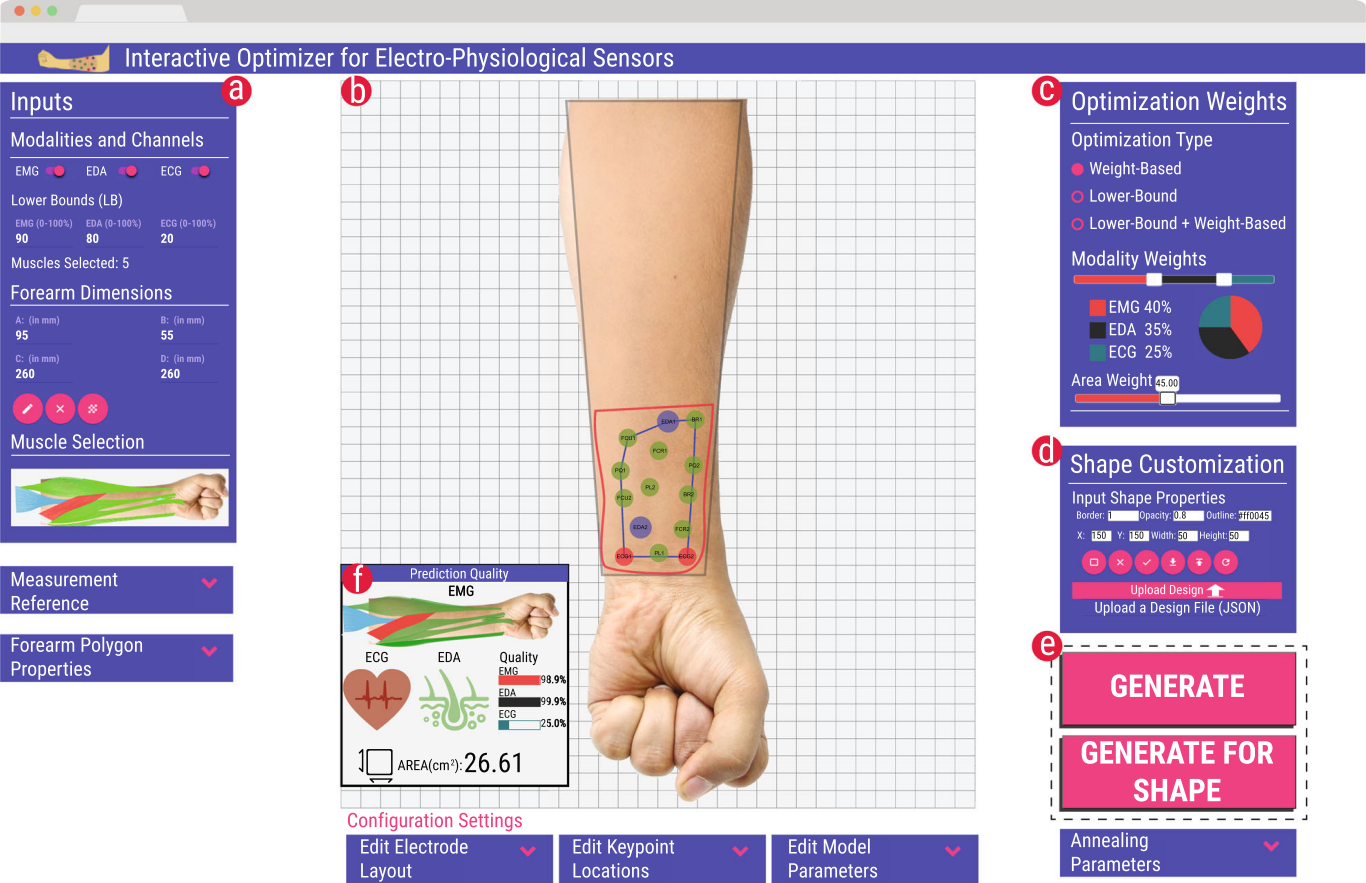
Screenshot of the graphical design tool for interactively generating and inspecting optimized results. **a** Input panel for selecting the modalities and muscles, setting forearm dimensions and setting the lower bounds. **b** The canvas area where the generated designs are visualized. Designs can be fine-tuned by drawing a desired location and shape or dragging individual electrodes. **c** Panel for choosing the optimization type, weights for each of the modalities, and overall sensor area. **d** Shape customization panel for fine-tuning the properties related to the sensor shape. Additionally, this panel also allows for uploading existing designs and exporting the current designs. **e** Buttons for one-click automatic generation of the layout. The result is displayed in real-time in the canvas area. **f** Panel visualizing quality metrics for the generated layout. Advanced functionality for use by experts can be accessed through drop-down panels. This includes functions for adjusting and editing the electrodes in the generated solution, adjusting the internal parameters of the model, tweaking the optimization parameters, and adjusting the appearance of the forearm polygon. The workflow for using the tool is shown in Supplement tool is shown in Supplementary Video 1.

A computational predictive model and optimizer are necessary but not sufficient for the rapid design of electrode layouts. To make the optimization approach accessible to a wide audience of practitioners and researchers and to ease visual analysis and rapid iterations of custom designs, an interactive software tool has been designed and implemented (Fig. 2). The graphical tool abstracts low-level details of the model, electrode design and optimization scheme (e.g., electrode sizes, spacing, placement, etc.), while exposing relevant parameters in an intuitive and user-friendly interface. It offers a Web-based interface that encapsulates the predictive model and automatically sets low-level parameters of the design. For instance, the size of electrodes is preset with appropriate dimensions for ensuring maximum performance, and their spacing is automatically adjusted by the optimizer. High-level parameters that allow for customizing the sensor can be adjusted through intuitive checkboxes and sliders. This offers a direct, fast, and user-friendly way of setting body dimensions, selecting the modalities the sensor will be able to capture (EMG, ECG, and/or EDA), and selecting specific muscles for EMG sensing. The sensing quality of one or multiple modalities can be easily prioritized by moving a slider. Similarly, the priority of a compact sensor vs. the highest possible sensing quality can be continuously adjusted. In our current implementation, the interface was designed for the anterior side of the forearm. However, it can be extended to support other body sites as well based on the underlying anatomical properties (e.g., muscles lines, types, and their directions, sweat gland concentration, etc.).

By default, the search space of the optimization scheme spans the entire surface of the body site. In cases when a more precise control over the location and shape of the sensor is required, the search space can be constrained interactively. As shown in Fig. 2 and video supplement (Supplementary Video 1), the location and shape can be quickly specified by sketching a free-form polygonal outline on the canvas, using a mouse or a touchscreen. This defines a region which the sensor design must not exceed. Lastly, more detailed settings can be adjusted in dropdown panels, if experts wish to do so. Then, with the click of a button, the sensor design is generated and optimized for the given parameters.

To allow for real-time visual analysis of the result’s quality, the design is immediately visualized within a few seconds, alongside metrics for the predicted quality of the sensor (Fig. 2b, f). If the design is not fully satisfactory yet, parameters can be fine-tuned and the design re-optimized. Moreover, a basic electrode layout editor has been incorporated, which enables the user to directly adjust the electrode positions if desired. The resultant quality metrics are immediately updated. These features are vital to enable a designer in-the-loop optimization^32,33^ approach: rather than simply accepting the solutions generated by the optimizer, the designer interacts in real-time with the optimizer; this allows for combining the strengths of algorithmic optimization with human creativity and knowledge of the application domain.

Finally, to ease sensor fabrication and ease replication, the generated design can be exported to a standard scalable vector graphics (SVG) file. This can be directly used for printing the electrode layout using conductive ink on a flexible substrate^6,34^. Alternatively, if off-the-shelf wet-gel electrodes are going to be used, the SVG file defines a stencil for electrode placement that is printed on a transparent PET. Holes can be punched through the PET film at electrode locations, and once overlaid onto the forearm, a marker can be used for marking electrode locations on the forearm. Electrodes can then be placed on these locations on the forearm. In addition, design solutions can be saved as a JSON formatted file for later use in the design tool. These functionalities help overcome a major drawback of the classic manual placement approach by making it possible to precisely replicate a specific electrode placement.

### Comparison of optimizer results with conventional designs

The performance of the optimizer was experimentally validated for tasks of various complexity. The experiment had two objectives. Firstly, understand how well a computationally optimized design performs in comparison to the standard placement techniques and an expert generated solution. The second objective was to assess the efficiency and scalability of the optimizer for more complex device configurations encapsulating electrodes that measure multiple physiological modalities.

To address the first objective, electrode layouts were optimized that capture one modality only. Electromyography was selected as the most demanding modality due to its strong requirements for precise electrode placement. A combination comprising three muscles was chosen, which together support a variety of arm movements^35^: Flexor Carpi Radialis (FCR), BrachioRadiali (BR), and Palmaris Longus (PL). The following electrode layouts were compared:

- BASELINE SOLUTION: This is a non-optimized rule-based solution generated following the existing placement guides for EMG electrodes presented in the literature^9,17,36,37^. Electrodes are placed at the respective muscle’s keypoints, which ensures the highest quality.
- QUALITY OPTIMIZED: The optimizer has traded-off size for achieving high-quality sensing. Details on how this solution was generated can be found in the “Methods” section.
- AREA OPTIMIZED: The optimizer has aggressively tried to reduce the size of the layout while trading-off sensing quality. Details can be found in the “Methods” section.
- EXPERT GENERATED: This solution was manually created by a human expert (a sports scientist, male, 31 years old, specializing in placing EMG electrodes for rehabilitation and performance monitoring with 6 years of professional experience). The expert was tasked to design a sensor layout for use near the wrist, ensuring the smallest possible size. The expert stressed the challenging nature of creating the design for multiple muscles in a compact form factor. The heuristic approach used by the expert was to first identify for each of the three muscles the muscle line and place the electrodes close to the wrist while ensuring the electrodes are approximately aligned with the muscle. Then, the expert aggressively reduced the inter-electrode distance while ensuring that there was at least a 10 mm distance between electrodes. He considered this minimum distance as absolutely essential to keep sensing quality at a reasonable level, which is in-line with recommendations presented in the literature^10^.

**Fig. 3:**
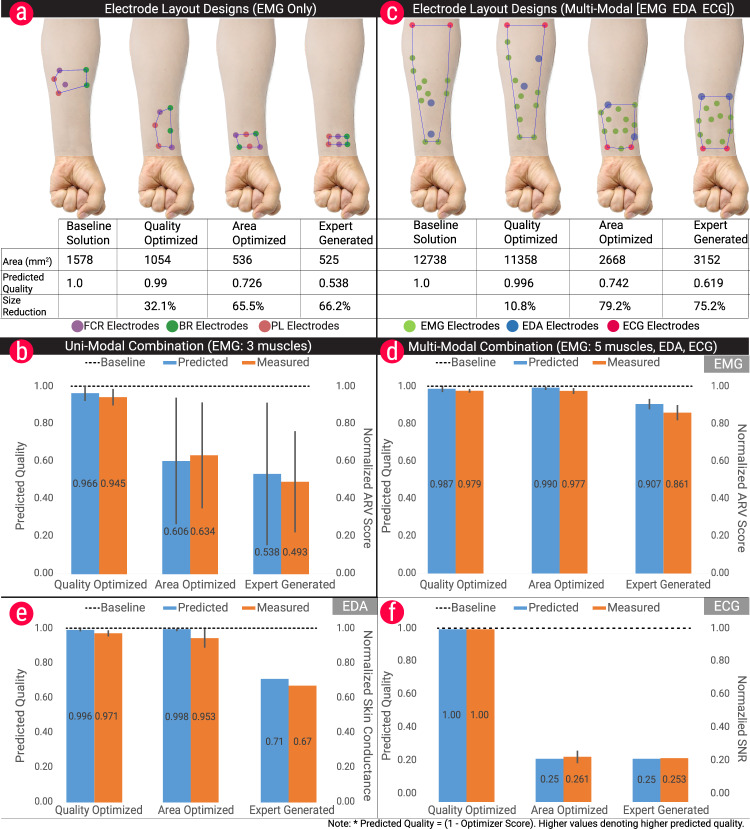
Comparison of the optimizer results with conventional designs and the experimentally collected physiological data. **a** Visual representation of the generated designs for the uni-modal combination, involving EMG with three muscles, alongside their area and quality score predicated by the optimizer (values are normalized w.r.t. the baseline solution). **b** Comparison of model prediction with empirically measured quality scores of EMG sensing. The model is able to accurately predict the sensing quality (values are normalized w.r.t the baseline solution). **c** Visual representation of the generated designs for the multi-modal combination, involving EMG with five muscles, EDA and ECG. **d**–**f** Modality-wise comparison of model prediction with empirically measured quality scores for EMG, EDA, and ECG sensing, showing the model accurately predicts the sensing quality. Note: The optimizer score ranges between 0 and 1, with 0 being the best. For better clarity, the graphs plot the complement value (1–Optimizer Score), which gives a direct measure of the quality predicted. Error bars indicate standard deviation.

Figure 3a depicts the generated designs alongside their area and quality score predicted by the model. (The optimizer score, i.e., the result of the cost function, represents the sensing quality predicted by the model. It is in the range [0, 1], with 1 being worst and 0 being the best. For better clarity, we report the quality score, which is the complement (1–Optimizer Score), with higher values denoting higher predicted quality.)

The BASELINE SOLUTION (predicted quality: 1.0) was taken as reference, and the scores for other solutions were normalized with respect to this condition. The QUALITY OPTIMIZED solution achieves a signal quality almost on-par with the BASELINE SOLUTION (average quality of 0.979 [max: 0.99, min: 0.96]), while considerably shrinking the sensor area by an average of 44% across the three participants (max: 56%, min: 33%). The AREA OPTIMIZED solution yielded a lower predicted sensor quality with an average of 0.60 (max: 0.72, min: 0.54) while however being able to shrink the sensor’s footprint to almost one-third of the baseline’s footprint (max: 65%, min: 48%). Noteworthy, it clearly outperforms the EXPERT GENERATED solution by offering a considerably higher predicted sensing quality (18% more) with only a minimally larger footprint (2% larger).

For achieving our second objective, we were interested in how the optimizer would perform for more complex combinations involving larger number of muscles and additional physiological modalities (EDA and ECG), resulting in a multi-modal sensor. To investigate the optimization of a multi-modal sensor, a complex combination was chosen, involving EDA and ECG modalities as well as EMG sensing for five muscles: Flexor Carpi Radialis (FCR), Brachiradialis (BR), Palmaris Longus (PL), Pronator Quadratus (PQ), and Flexor Carpi Radialis (FCU). It involves placement of 14 measurement electrodes for acquiring signals. Arranging all these electrodes while ensuring a minimum size is a very taxing task, even for experts.

Similar to the uni-modal case described above, four electrode layouts were compared: a BASELINE solutions that is not optimized and follows the existing placement guides for EMG^9,17,27^, EDA^15^, and ECG^16^ electrodes presented in the literature; a QUALITY OPTIMIZED design; an AREA OPTIMIZED design; and an EXPERT GENERATED design. Figure 3c depicts the generated designs alongside their area and quality score predicted by the model. The average reduction in the area for a QUALITY OPTIMIZED solution was 10% (max: 14.4%, min: 4.8%), with an average drop in quality of only 0.5%. This reduction is smaller compared to the uni-modal case presented above due to specifics of ECG sensing: the ECG key points located closer to the elbow on the upper forearm produce higher signal quality, whereas the quality decreases drastically closer to the wrist. Therefore the optimizer favors designs that span a larger area up to the forearm. The average reduction in area for the AREA OPTIMIZED solution was 75.9% (max: 79.1%, min: 68%) with an average reduction in quality of 26.2% (min: 25.6%, max: 26.7%). The AREA OPTIMIZED solution again clearly outperformed the EXPERT GENERATED design (75% reduction in size with 38% drop in quality), yielding a comparably smaller footprint while offering considerably higher predicted sensing quality than the EXPERT GENERATED design. The quality for EMG was high for all the solutions since there was enough space for electrodes to be aligned to their respective muscle lines while maintaining a good inter-electrode distance. For EDA, the key takeaway here is that the optimizer scores were very similar between AREA OPTIMIZED and QUALITY OPTIMIZED solutions, owing to the fact that a similar number of sweat glands were covered in both the AREA OPTIMIZED and QUALITY OPTIMIZED solutions. For ECG, the position of the electrodes was the same for the BASELINE SOLUTION and the QUALITY OPTIMIZED solution; therefore, the difference in the SNR levels across the designs was minimal. However, for the AREA OPTIMIZED solution, the quality drops drastically since the electrode locations are located further below on the forearm.

### Experimental validation of prediction quality

To experimentally validate the optimizer’s prediction quality for uni-modal optimization, EMG data were recorded for each muscle on each design. Three volunteer participants (two female, one male, mean age: 8 years old, SD: 2.9) were recruited for the experiment. The physical measurements of the forearm were procured from the participants. QUALITY OPTIMIZED and AREA OPTIMIZED designs were generated for the participants’ arm dimensions through the software tool. The EXPERT GENERATED design was manually generated for only one participant. Wet-gel electrodes (Kendall™ Covidien, H135SG, Sensor Area: 50 mm^2^) were placed on the participants’ right forearm at the locations specified in the design. Wet-gel electrodes are the experimental standard for measuring physiological signals and provide a stable baseline for evaluating the predicted signal quality of the optimizer. In the Supplementary Information (Fig. 9), our results indicate that the quality prediction of the optimizer also generalizes to dry electrodes fabricated through conductive inkjet printing, demonstrating a close agreement between the predicted and measured signal quality.

The EMG signals were average rectified. The peaks corresponded to the signal when there was a muscle movement. For each muscle, the mean Average Rectified Signals (ARVs) were calculated across all the trials. As shown in Fig. 3b the scores predicted by the optimizer match very closely with the experimentally measured values. Overall, there was an average 2% difference between the predicted and measured values across all the muscles and all the participants for the QUALITY OPTIMIZED condition. The difference is marginally higher for the AREA OPTIMIZED solution (2.8%) and for the EXPERT GENERATED solution (4.5%). These results show that the optimization scheme can closely predict the sensing quality of a real sensor and offers an effective way of generating highly compact designs while maintaining a high-quality signal acquisition capability.

To experimentally validate the optimizer’s prediction quality for multi-modal sensors, EMG, EDA and ECG data was recorded for each of the layout conditions. Wet-gel electrodes (Covidien, H124SG) were placed on the participants’ right forearm at the locations specified in the design. Similar to the Uni-Modal combination, the EMG signals were average rectified. For the EDA, skin conductance measurements were obtained through off-the-shelf GSR sensors by placing the electrodes on the fingertips. Finally for the ECG measurements, a commercial portable ECG device (EKG Monitor MD100E, ChoiceMMed) was used for recording. For details on the method and the raw signals please see “Methods” section and Supplementary Information respectively.

The experimentally measured value for EDA and ECG are skin conductance level and SNR values, respectively. The SNR values as reported in prior work^16^ correspond to the ratio of the QRS wave peak-to-peak voltage to the T-P wave peak-to-peak voltage. For EMG, the difference between measured and predicted values across all participants and all muscles was 0.8% for the QUALITY OPTIMIZED layout and 1.3% for the AREA OPTIMIZED layout. For EDA signals, the average difference between the predicted and measured values was 2.5% for the QUALITY OPTIMIZED layout and 4.5% for the AREA OPTIMIZED layout.

**Fig. 4:**
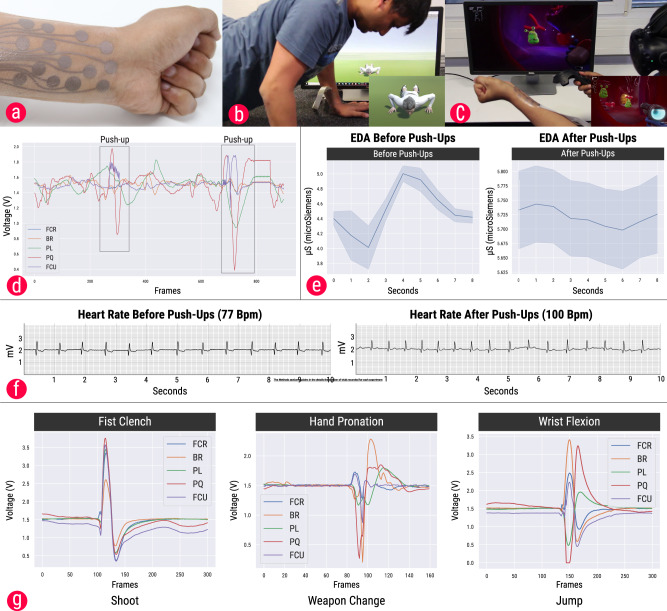
Example applications. **a** Ultra-thin temporary tattoo with compact sensor layout generated by the optimizer and fabricated with an off-the-shelf desktop inkjet printer. **b** Augmented reality exercising application: a virtual character performs push-up motion when the user performs a push-up. **c** A virtual reality game in which EMG-sensed gestures are used for controlling the virtual character in a first-person shooter game. **d** Raw signals of the EMG signals when performing a push-up exercise. **e** Increase in the skin conductivity levels before and after the push-ups. The shaded region represents the standard deviation. **f** Difference in the heart rate before and after performing the push-ups. **g** Raw EMG signals of the five muscles for each of the gestures used in the virtual reality game.

The average difference in measured skin conductance levels between the BASELINE SOLUTION and QUALITY OPTIMIZED solutions was 0.0776*μ*S (resulting in an average of 2.9% difference) and 0.1096*μ*S (resulting in an average of 4.7% difference) for the BASELINE SOLUTION and AREA OPTIMIZED solution. These differences are in-line with the variance found in skin conductance levels on the forearm as reported in prior work^15^. For ECG, the difference in the predicted and measured values for the AREA OPTIMIZED and EXPERT GENERATED designs was very small as well (1.1% and 0.3%, respectively). It should also be noted that, although the quality of ECG signals drops drastically near the wrist, the distinct QRS peaks can still be noticed, implying the signal can be used for measuring the beats per minute (BPM) or heart rate variability (HRV) (Fig. 4e).

### Applications

The computational design approach and the optimizer are generic. The generated designs can be implemented with either commercial gel electrodes or dry electrodes fabricated with conductive materials. Two application cases have been realized to demonstrate the benefits of the proposed approach for applications of electrophysiological sensing beyond the medical field, such as interactive sports devices, gaming, and virtual reality. Applications in these areas benefit from devices that have a small footprint while capturing multiple biosignals. Moreover, they impose high demands on ergonomic wearability to not obstruct body movement. These requirements can be met by integrating the computational design approach with a rapid fabrication technique^19,34^ to realize compact layouts of dry electrodes on ultra-thin temporary tattoo films.

To demonstrate an end-to-end pipeline for iterative design and rapid prototyping, a conductive inkjet printing technique^34^ has been coupled with the design tool. This combination has been utilized for fabricating an ultra-thin temporary tattoo device encapsulating EMG, EDA, and ECG electrodes. The AREA OPTIMIZED device (Fig. 3c) was fabricated on temporary tattoo paper for measuring EMG, EDA, and ECG signals (Fig. 4a). Once the design was generated by the tool, a standard vector graphics application was used for creating the routing traces to connect the sensor to an external microcontroller. A flexible printed circuit (20 pins, 1 mm pitch) was connected to the device with the help of a conductive z-axis tape which in turn was interfaced to two Arduino microcontrollers. One microcontroller (Arduino Uno, ATmega328P) was used to interface the five EMG channels, while another microcontroller (Arduino Uno, ATmega328P) unit interfaced with the EDA and ECG channels. Details of the hardware specifications can be found in the “Methods” section.

Recording of physiological signals can be beneficial for personal health analytics. Inspired by new opportunities for improving physical exercising with augmented reality, an application for augmented push-up exercising has been developed. In this application, a virtual on-screen avatar performs push-ups along with the user and offers a synchronized experience using biosignals (Fig. 4b, Supplementary Video 3). When the user performs a push-up, the movement is detected through the EMG signals picked up through the temporary tattoo device on the wrist. A custom Unity application loads the virtual avatar and processes the EMG signals. When the signal corresponding to the PQ muscle exceeds a threshold, a push-up is recognized (Fig. 4d). Then, the push-up counter is incremented, and the virtual avatar performs the push-up. The EDA and ECG signals can also be monitored. Figure 4f shows the change in the heartbeat before and after performing five push-ups, while Fig. 4g shows an increase in the skin conductance levels after performing push-ups. The computational design approach integrated with the custom fabrication pipeline enables the rapid design of a compact epidermal interface that is ergonomic to wear during physical movement. Future designs could involve designs placed at various other body locations, such as the biceps, to monitor multi-modal physiological signals while performing physically demanding activities.

The use of physiological signals in augmented or virtual reality environments is actively explored in research^38^. Inspired by this, we developed a second application case demonstrating that a multi-modal sensor tattoo designed with the optimizer can be used as an intuitive body-based controller for gestural input in virtual reality applications. A virtual reality first-person shooter game was implemented in Unity; the interaction with the game was integrated through hand gestures that can be recognized through EMG signals. In the game, the user has to explore and shoot all the germs present in the human body (Fig. 4c, Supplementary Video 3). Three gestures were recognized in real-time through thresholding of the signals from five EMG channels: a “Fist Clench” gesture is used to shoot a given target; a Hand pronation gesture is used to change the weapon, and a wrist flexion gesture is used for jumping. The minimally invasive form factor of the multi-modal patch can peripherally record the biosignals without the need for dedicated sensors at multiple locations on the body. While in this scenario, we have demonstrated the use of EMG as a medium for gestural interaction, recording multi-modal physiological data can open up new possibilities for interaction and experiences in the context of augmented and virtual reality. For example, the EDA and heart rate variability data could be used for detecting the mood of the user and adapt the game’s content on the fly.

## Discussion

The results reported in this article demonstrate the feasibility and effectiveness of computationally designing and optimizing multi-modal electrophysiological sensor layouts. Using a computational design paradigm coupled with an optimization-based approach paves the way for automatically generating highly compact wearable devices that can monitor multiple electrophysiological modalities. With an integrated predictive model that takes into account the human anatomy, the electrode design task has been formulated as a geometric packing optimization problem. A Web-based graphical software tool allows for interactively specifying desired design parameters in a user-friendly way and for visually analyzing the quality of generated designs. Results from the experimental evaluations show that the generated designs outperform expert-generated solutions and can considerably reduce the size of a device. Multi-modal sensors can be reduced in size by up to 79% when compared to the BASELINE SOLUTION. The sensors are also considerably smaller (19.5%) than the EXPERT GENERATED design, which suggests that the approach can create solutions that provide a very good balance between signal quality and size. Similarly, for uni-modal sensors, the AREA OPTIMIZED solution is only marginally larger (2%) but achieves considerably better quality (18.2%). The results further demonstrate high quantitative agreement between experiments and the model predictions. Two application examples were implemented and showed the feasibility of an end-to-end pipeline for computational design and fabrication of compact and ergonomic wearable sensing devices. The computational design approach is scalable to other electrophysiological modalities provided, and there exists an empirically validated model that defines the placement of electrodes.

This proof-of-concept study is subject to several limitations that open a series of perspectives for future research. The model and tool are currently limited to one body location—the anterior side of the forearm. High-quality clinical-grade acquisition of ECG and EDA biosignals is usually performed on the chest and fingertips. However, the forearm offers the benefit of superior wearability (wearability in design research is defined as the physical shape of wearables and their active relationship with the human form^24^). The forearm is one of locations that are most unobtrusive for wearable objects^24,39^ and offer unmatched opportunities for user interaction—important benefits when considering highly practical non-medical applications such as entertainment computing, human–machine interaction, and wearable computing. While the methods presented here are expected to generalize to other body locations where continuous models are available (e.g., on the chest where continuous ECG models are available, along with placement strategies for a few muscles), there still remain several challenges to be addressed: (1) To the best of our knowledge, there exists no continuous model that evaluates ECG signals on the forearm. The discrete model used for ECG mapping in our study is simplified. Of note, this is not a limitation of the method; more advanced continuous models for ECG signals on the forearm and on other body locations should be integrated in future studies. (2) A variety of parameters, including subcutaneous fat levels, skin moisture levels, and variations in skin–electrode contact, all affect the sensing quality^40,41^. While the currently existing models do not consider these factors, it can be observed that the model predictions closely match the experimental measurements that were taken in the real world. It will be important for future work to develop more sophisticated models that capture more of these factors, most notably sub-cutaneous fat levels. (3) Future more advanced models could integrate additional metrics for EMG, such as RMS (Root Mean Square), Conduction Velocity, and Frequency Response. These could be beneficial for specific applications such as gait analysis, fatigue analysis, etc. Future implementations also should expand the scope of computational design and optimization to additional electrophysiological modalities, such as EEG and EOG. (4) Currently, our model is agnostic of the type of electrode. Different electrode types can affect the signal quality due to differences in impedance, durability of tight skin contact, or effects of skin moisture, amongst others. While the dry electrodes fabricated through our technique have low impedance and offer tight skin contact, they need to be studied more extensively with respect to the rate of degradation of the skin contact and impedance levels over an extended duration. These factors are crucial and generic for all types of dry electrodes, which can be realized through various fabrication strategies. While evaluating multiple types of dry electrodes is beyond the scope of this work, this first study provides evidence that computational design approaches can be integrated with custom-fabricated dry electrodes.

For all these modalities requiring precise placement of electrodes on the body, this computational approach could pave a promising way for guiding electrode placement and reducing manual placement overhead. From an optimization perspective, our current implementation is based on simulated annealing, which needs to be stopped after a finite number of iterations without exactly knowing how far the result is from the optimum. One approach to improve the optimization scheme in future work is to use a mixed-integer optimization that yields a rigorous lower bound on the signal quality using methods such as branch-and-bound that could serve as a benchmark.

Considering that electrophysiological sensing is becoming more widespread and is making its way into non-medical disciplines, approaches based on computational design, rather than manual heuristics for experts, promise to accelerate the widespread adoption of these sensing techniques. This first exploration unfolds a new dimension for the design of electrophysiological sensors leveraging the power of computational optimization, guided by an interactive real-time design tool. This can represent a significant step towards a fully automated and highly scalable pipeline for the design and creation of electrophysiological sensing devices.

## Methods

### Predictive model for EMG electrodes

Given a set of forearm measurements (Supplementary Fig. 1) *F* = {*f*_1_, *f*_2_, *f*_3_, *f*_4_}, the five muscle lines can be reconstructed based on the guides from prior work^9,17,27^. Based on this, a set of keypoints is calculated which is represented by *K*_*EMG*_ = {*k*_1_, *k*_2_, *k*_3_…, *k*_*ℓ*_}. These keypoints consist of ideal locations for EMG electrode placement. For EMG acquisition, the electrodes should have a minimum surface area of 50 mm^2^ and the diameter should not exceed 10 mm^9,10,28^. To ensure good signal acquisition, the model incorporates electrodes that have a surface area of 50 mm^2^.

For a given pair of electrodes that measure the potential of a specific muscle, a series of pre-checks are made. Firstly, both electrodes need to be within a distance of 1 cm from the muscle line. This is based on the recommendation from prior work, which suggests that more than 1 cm offset from the muscle line could result in a considerable decrease in signal and recognition accuracy^42^. If at least one of the electrodes is farther away, then a score of 1 is assigned. Otherwise, an additional check is made to ensure that both electrodes are not present within innervation zones. Based on the recommendations from Barbero et al.^17^, innervation zones (IZ) are unsuitable locations to place electrodes. Hence if either of the electrodes falls within these regions, then a score of 1 is assigned. The innervation zones for muscles are well documented in the literature^17,36^.

Following successful checks for these conditions, a normalized score is calculated based on the electrode orientation with respect to the muscle line and the inter-electrode distance. The orientation of the electrodes with respect to the muscle line is calculated as follows:

4 $$\theta =\arccos \left(\frac{\overrightarrow{{k}_{i}}\cdot \,\overrightarrow{e}}{| \overrightarrow{k}| \cdot | \overrightarrow{e}| }\right)$$

where $\overrightarrow{k}$ is the vector connecting the two keypoints for the specific muscle *m* in the set *K*_*EMG*_ (this is the vector representing the muscle line) and $\overrightarrow{e}$ is the vector connecting the two measuring EMG electrodes $e^{\prime}$ and *e″*.

Once the angle between the muscle line and the electrodes is determined, data from the literature is used to inform the model. Merletti et al.^43^ showed how the quality of the EMG signal is affected by the orientation between the muscle line and the electrodes, and showed that the signal drastically drops with misalignments >60 degrees. The least squares curve-fitting method has been used to derive the closest curve (*R*^2^ = 0.9971), which fits the data presented in prior work^43^. Based on this, the energy function for the orientation of the electrodes is defined by:

5 $$\omega (\theta )=\left\{\begin{array}{ll}0.0057\theta +0.000181{\theta }^{2}&\,{{\mbox{if}}}\,\ \theta \le 6{0}^{\circ }\\ {\hskip -82pt}1,&\,{{\mbox{otherwise}}}\,\end{array}\right.$$

If the electrode orientation is <60^∘^, then the inter-electrode distance $| \overrightarrow{e}|$ is calculated for the electrode pair. The model is informed from prior literature, which shows how the signal varies with respect to changes in the inter-electrode distance^43^. The data were normalized and a best-fitting curve was calculated using the method of least squares (*R*^2^ = 0.9986). The energy function for the inter-electrode distance $| \overrightarrow{e}|$ for an electrode pair is as follows:

6 $$\nu (| \overrightarrow{e}| )=\left\{\begin{array}{ll}\max (0,1.0125-0.0586| \overrightarrow{e}| +0.0007| \overrightarrow{e}{| }^{2})&\,{{\mbox{if}}}\,\ 5 \, < \, | \overrightarrow{e}| \le 25\\ {\hskip -165pt}0&\kern0.5pc {{\mbox{if}}}\,\ 25 \, < \, | \overrightarrow{e}| \le 60\\ {\hskip -165pt}1&\kern-1pc{{\mbox{if}}}\,\ 60 \, < \, | \overrightarrow{e}| \end{array}\right.$$

Prior literature^10,44^ suggests that large inter-electrode distances (> 60 mm) can create a drastic drop in the signal quality. Hence a limit of 6 cm was applied to ensure that large inter-electrode distances were not generated.

For calculating the overall score of the EMG electrodes, the inter-electrode distance and electrode orientation are taken into account. Combining Eqs. (5) and (6), the overall energy function for a muscle *m* is calculated as a weighted average of the angle orientation score and the inter-electrode distance score, which is defined as:

7 $${O}_{1i}=\left\{\begin{array}{ll}\alpha \cdot \omega ({\theta }_{i})+(1-\alpha )\cdot \nu (| {\overrightarrow{e}}_{i}| )&\,{{\mbox{if}}}\,\ {e}_{i}^{\prime},{e}_{i}^{{\prime\prime} }\,\notin\, {R}_{i}\\ {\hskip -105pt}1&\kern-0.5pc{{\mbox{otherwise}}}\,\end{array}\right.$$

where *α* and 1 − *α* are the priorities assigned for both parameters and serve as the calibration parameter for EMG measurement hardware. *R*_*m*_ corresponds to the innervation zones which, are not suitable locations for the electrode placement. In the current model, equal priorities (*α* = 0.5) are given for both these factors.

For *n* selected muscles, the overall EMG score is then calculated as the product of the EMG weight assigned and the average of the overall muscle scores of each of the selected muscles, which can be formulated as:

8 $${O}_{1}(F,{E}_{1},{w}_{1},S)={w}_{1}\cdot \frac{1}{m}\mathop{\sum }\limits_{i=1}^{m}{O}_{1i}$$

where *F* = {*f*_1_, *f*_2_, *f*_3_, *f*_4_} is the set of forearm measurements, *E*_1_ is the electrode set for EMG, *w*_1_ is the weight determining the priority for EMG in the overall objective function, and *S* is the optional input shape.

### Predictive model for EDA electrodes

Electrodermal activity (EDA) measures the changes in electrical conductance of the skin that result from sympathetic neuronal activity. The EDA activity monitors the sweat gland activity. A sensor typically consists of two electrodes placed on the body, between which the conductance is measured. Based on the recommendations from the literature^45^, the surface area of electrodes was set to 0.78 cm^2^. The quality of a given layout of electrodes for sensing EDA is based on two factors: the density of the sweat glands at a given body location and the inter-electrode distance.

### Electrode location on the body

The electrodes for EDA response can be placed on various locations on the body as long as a required minimum number of sweat glands are captured. The density of sweat glands varies across the body, with higher concentrations present at fingertips, palms, and forehead. The density of sweat glands is rather a discrete function, and the forearm is reported to have about ≈ 108/*cm*^2^^46^. The sweat gland concentration for various other locations is reported in the literature^46,47^. Prior work has shown that a minimum number of sweat glands that need to be covered between EDA electrodes for maintaining functionality is 140^48^.

For two given circular electrodes, the area covered between two electrodes comprises the surface of the electrodes and the area comprised in-between electrodes, as shown in Supplementary Fig. 2 . Hence, if *D*_*s*_ is the density of sweat glands at a specific location, the number of sweat glands covered by the electrodes is given by: *N*_*s*_ = (*πr*^2^ + (*d* × 2*r*)) × *D*_*s*_.

### Inter-electrode distance

The skin conductance level is linearly proportional to the number of sweat glands between the electrodes. This is because the glands act as resistors connected in parallel, thus bringing the skin resistance down^49^. The recommended distance between the electrodes is 5–6 cm^15,49^. For larger distances, the two electrodes risk not being on the same dermatome, which can lead to invalid readings^49^. Assuming an ideal inter-electrode distance of 6 cm, the maximum number of sweat glands that can be covered on the forearm is ${N}_{\max }=(\pi 0.{5}^{2}+(6\times 2\times 0.5))\times 108\,$ ≈ 733 sweat glands.

The energy function is as follows:

9 $${O}_{2}({E}_{2},{D}_{s})=\left\{\begin{array}{ll}{\hskip -47pt}1&\,{{\mbox{if}}}\,\ {N}_{s}\le 140\ \,{{\mbox{or}}}\,\ {d}_{s} \, > \, D\\ 1-{N}_{s}/{N}_{\max }&\kern-4pc{{\mbox{otherwise}}}\,\end{array}\right.$$

where *d*_*s*_ is the inter-electrode distance and *D* is the recommended distance of 6 cm. *N*_*s*_ is the number of sweat glands covered by the electrodes for a given inter-electrode distance *d*_*s*_ and ${N}_{\max }$ is the maximum number of sweat glands that can be covered on the forearm for a recommended distance of 6 cm (which is ≈733). For inter-electrode distances larger than 6 cm, a score of 1 is assigned since larger inter-electrode distances are not recommended.

#### Predictive model for ECG electrodes

Electrocardiogram refers to the recording of electrical changes that occur in the heart during a cardiac cycle. It works on the principle that a contracting muscle generates a small electric potential that can be detected and measured through electrodes suitably placed on the body. Clinically, the measurements are obtained by placing 12 electrodes near the chest^18^. However, more recently, for ambulatory and wearable devices, three-electrode configurations have been explored^16,23,50^.

This approach is based on prior work, which designed a three-electrode ECG configuration on the forearm^16,21,23,30^. Based on prior work^16^, a set of keypoints (near the wrist, upper forearm, and the central region of the forearm) on the forearm were chosen as the ECG locations. For each of these locations, the ECG recordings were obtained with a portable commercial ECG device (EKG Monitor MD100E, ChoiceMMed). For each of the recordings, the SNR values were computed as follows based on prior work^16^:

10 $$SNR=\frac{(QRS)EC{G}_{p-p}}{(T-P)nois{e}_{p-p}}$$

where the *ECG*_*p*−*p*_ is the peak-to-peak ECG QRS amplitude, and the *noise*_*p*−*p*_ is the peak-to-peak noise amplitude from the T-P interval. The SNR values for each of the combinations are shown in Supplementary Fig. 3. For optimization, the SNR values are normalized with respect to the highest achieved SNR. The ECG electrode pair on the upper forearm achieves an optimizer score of 0, indicating that this is the best electrode pair, while the electrode pair near the wrist achieves a score of 0.75 because the SNR levels at this location drop drastically by about three-quarters.

For a given set of keypoints *k*_1_ and *k*_2_, the energy function is given as:

11 $${O}_{3}({E}_{3})=\frac{SNR(k^{\prime} ,k^{\prime\prime} )}{SNR({k}_{max}^{\prime},{k}_{max}^{^{\prime\prime} })}$$

where *SNR*(*k*_1*max*_, *k*_2*max*_) is the SNR for the keypoints, which can provide the maximum SNR levels on the forearm, and *SNR*(*k*_1_, *k*_2_) is the SNR for a given set of keypoints.

#### Predictive model for area

For a given layout, the area of the convex hull of all electrodes is calculated based on the Graham scan algorithm^51^. This area is then normalized with respect to the area of the BASELINE SOLUTION for a given combination of modalities. While we have assigned a linear cost penalty for the area, a quadratic cost penalty can be assigned alternatively to more aggressively shrink the sensor size. The energy function is as follows:

12 $${O}_{4}(E)=\frac{A(E)}{A({E}_{b})}$$

where *A*(*E*) is the area of a given layout, and *A*(*E*_*b*_) is the area of the BASELINE SOLUTION.

#### Lower-bound-based optimization

In addition to the weight-based optimization scheme in which relative weights are provided for the EMG, EDA, and ECG modalities, we additionally implemented a lower-bound-based optimization scheme.

To this end, we increase the objective function by a penalty for each modality that grows exponentially with the extent of the violation of the corresponding lower bound.

That is, we define

13 $${P}_{k}:= p\cdot ({e}^{\max ({O}_{k}-{\ell }_{k},0)}-1)$$

where *ℓ*_*k*_ is the specified lower bound for modality *k* and *p* is a parameter to control the softness of the lower-bound constraints. The higher the value of *p* is, the harder the lower bound is enforced by the optimizer. Observe that a penalty only occurs if a lower bound is violated, i.e., *P*_*k*_ = 0 whenever *O*_*k*_ ≥ *ℓ*_*k*_.

In the hybrid scheme, the complete objective function becomes

14 $$O(F,W,S)=\mathop{\sum }\limits_{k=1}^{3}({w}_{k}\cdot {O}_{k}+{P}_{k})+{w}_{4}\cdot {O}_{4},$$

whereas *w*_1_ = *w*_2_ = *w*_3_ = 0 when we only consider the lower bounds.

#### Inputs and constraints

The optimization step requires a set of user inputs, such as the forearm dimensions, modality weights, and an (optional) input shape, which serve as parameters and constraints for the integrated predictive model.

#### User inputs

The inputs to the model which are provided through the software tool are formalized as follows:

- Dimensions of the location: The dimensions refer to the body site (the forearm in our implementation).
- Modalities: These involve the selection of desired modalities.
- Individual muscles: These involve the set of muscles for EMG sensing.
- Area weight or outline of sensor shape: The shape of the sensor layout can be sketched by the user. Alternatively, if no shape is specified, the tool automatically generates the appropriate sensor layout based on the weight provided for the small area.
- Weights: These include the weights for each of the modalities. These weights can be represented as *W* = {*w*_1_, *w*_2_, *w*_3_, *w*_4_} where *w*_1_ + *w*_2_ + *w*_3_ = 1 and correspond to the weights of EMG, EDA,, and ECG respectively. *w*_4_ refers to the weight for the Small Area of the sensor layout.

#### Derived parameters

Based on the user inputs, the following parameters are derived:

- Keypoint set: Given the dimensions, modalities, and the muscle selection, the keypoints set *K* = {*k*_1_, *k*_2_, *k*_3_…, *k*_*n*_} is calculated. These keypoints consist of ideal locations for EMG and ECG electrode placement.
- Electrode sizes: Based on the selected modalities, the optimizer selects the size of electrodes for high-quality signal acquisition. The electrode sizes were fixed as follows: 50 mm^2^ for EMG and ECG electrodes and 80 mm^2^ for EDA electrodes. These sizes were chosen such that they match the dimensions of electrodes that are commercially available. Finally, prior literature also suggests that increasing the electrode surface area does not necessarily result in better signal quality^10,45^.
- Electrode set: Based on the above three parameters, a measuring electrode set *E* = {*e*_1_, *e*_2_…, *e*_*ℓ*_} is generated, which contains disjoint subsets of electrodes ${E}_{1}=\{{e}_{1}^{\prime},{e}_{1}^{{\prime\prime} },\ldots ,{e}_{m}^{\prime},{e}_{m}^{{\prime\prime} }\}$ for EMG, *E*_2_ = {*e*_2,1_, *e*_2,2_} for EDA, and *E*_3_ = {*e*_3,1_, *e*_3,2_} for ECG, i.e., ${E}_{1}\dot{\cup }{E}_{2}\dot{\cup }{E}_{3}\subset E$. For all these electrodes, a maximum of two reference electrodes are required: one electrode which acts as a common reference for the EMG and one electrode which is required for the ECG. Both these electrodes need to be placed away from the forearm (preferably near the shoulder/chest) for having a high-quality ECG signal.

For ensuring the validity of the generated electrode layout, the following set of constraints have been imposed:

- Overlapping electrodes: To ensure that no electrodes overlap with each other, the center-to-center distance between each pair of electrodes with radii *r*_1_ and *r*_2_ must be greater than *r*_1_ + *r*_2_. For ensuring a safe distance between all the electrodes, the pair-wise inter-electrode distance between all pairs of electrodes was set to at least 12 mm. To ensure that all the electrodes within a layout are inside the input region sketched by the user, the Point-in-Polygon (PIP) algorithm^52^ was implemented. For all the generated solutions, this constraint is checked and only if it is met, the energy of the layout is calculated.

#### Validation of optimizer

The key goal of the experiment is to demonstrate that the optimizer generates valid and functional solutions. We were also interested in the broad spectrum of solutions that could be generated. Therefore the entire forearm space was sampled, allowing for (1) informing about the influence of the search space on the quality of the generated solutions and (2) providing a wide range of solutions with varying levels of quality and sizes sampled across the entire forearm search space. Note that in typical usage scenarios, it is not required to sample the entire forearm space; instead, an optimal solution can be directly generated by setting the desired priority for a small area or by providing a desired shape of the sensor.

The entire forearm was sampled at high resolution, starting at the wrist. Two configurations were chosen: a multi-modal configuration where all the modalities were included (five muscles, EDA, and ECG) and a uni-modal configuration with EMG only (three muscles). Starting at the wrist, the search space for the optimizer was incrementally increased by providing a bounding box as shown in Supplementary Fig. 4. The height of the bounding box was increased in 1 mm increments until the box covered the entire forearm, as shown in Supplementary Fig. 4. For each 1 mm increment, a solution was generated through the optimizer. The AREA OPTIMIZED solution was identified as the first solution that gives an optimizer score lower than 1. For obtaining the QUALITY OPTIMIZED solution, the search space was incrementally decreased in 1 mm intervals, starting at the top of the forearm until the predicted signal quality dropped below 0.9. The QUALITY OPTIMIZED solution was then identified as the solution which had the smallest size out of all solutions that have predicted quality of ≥0.95, or ≤0.05 optimizer score. The annealing parameters were kept constant for all iterations. Each iteration generated a design file which contained information about the electrode layout, quality, area, and other configuration information. For the multi-modal configuration containing EMG, EDA, and ECG modalities, there were a total of 193 iterations, with each iteration picking an optimal solution from a set for 15,490 randomly generated solutions, resulting in a total of 2,989,570 solutions. For the uni-modal combination involving three muscles, a total of 122 iterations were generated, resulting in a total of 1,889,780 solutions.

The smallest possible solution (AREA OPTIMIZED) for the uni-modal configuration was generated at a window of height 3 cm. No solution was generated below this height since there was not enough space for the optimizer to fill all the electrodes. The solution generated by the optimizer was slightly larger in size than the EXPERT GENERATED solution because of the constraint imposed, which limits too small inter electrode distances (the inter electrode distances between all pairs of electrodes is kept at least 12 mm). It is noteworthy that despite this constraint, the optimizer was able to shrink the device size to a level that is comparable to the expert-generated design.

The window height was 7.8 cm for the multi-modal combination. The relatively large window height was required due to the fact that the multi-modal case requires a larger number of electrodes (14 electrodes) than compared to the uni-modal configuration (six electrodes). It should be noted that these window heights depend on factors such as the configuration chosen, the number of electrodes to fit in, and the individual forearm dimensions.

#### Experimental data collection

Commercial gel-based electrodes (Kendall™ Covidien H135SG, Sensor Area: 50 mm^2^ for EMG and ECG^53^, Kendall™ Covidien H124SG, Sensor Area: 80 mm^2^ for EDA^54^) were used to experimentally evaluate the performance of the optimization technique.

#### EMG data collection

The primary functions of each of the muscles were identified from the literature. For each of the muscles, participants were instructed to perform maximal voluntary contractions with five repetitions. Before the start of the experiment, the participants were free to perform and practice the contractions. EMG recordings were recorded using a custom hardware acquisition unit (see the section on “Hardware Interfacing”). Digitized signals were full-wave rectified and integrated to calculate the Average Rectified Value (ARV). For each of the muscles, the movements performed for EMG signal capturing are described in Table 1.

**Table 1:** Five muscles used for the experimental condition and their corresponding voluntary contraction identified from literature^35^.

Muscle	Voluntary muscle contractions
Flexor Carpi Radialis (FCR)	The forearm was rested on a table; elbow slightly turned inward; palm upward. Wrist flexion was performed at maximal contraction level^60^.
Brachioradialis (BR)	The elbow was flexed to 90°. Then movement was performed from full pronation to neutral^61^.
Palmaris Longus (PL)	The forearm was rested on a table with the wrist in neutral position. Standard hypothenar abduction (maximal contraction) was performed^62^.
Pronator Quadratus (PQ)	The elbow was flexed to 90° in mid-air; the wrist was closed to form a fist. The movement was performed from full pronation to neutral^63^.
Flexor Carpi Ulnaris (FCU)	The forearm was rested on a table; elbow slightly turned inward; palm upward. Wrist adduction was performed at maximal contraction level^64^.

#### EDA data collection

For EDA, the participant underwent a Stroop color test^55^. This test has been used in prior work for assessing EDA response^19^. In brief, cognitive stimuli were presented to the subject through the use of words of different colors which were either conflicting (word and color of text were different, e.g., “blue” was written in green color) and non-conflicting (word and color of text were the same). The participant was instructed to state the color of the word and not read the text. The task consisted of an initial 1 min rest period followed by a 2–3 min long Stroop test. This was followed by a final 1 min rest period. The reference skin conductance level was also measured for each of the conditions by placing a commercial EDA sensor consisting of dry metallic electrodes (Seeed Studio Groove^56^) on the fingers. One electrode was placed on the index finger, while the other electrode was placed on the middle finger.

#### ECG data collection

For ECG signal acquisition, the participant was at rest, with the hands on a table, while a commercial portable ECG device (MD100, ChoiceMed) logged the data for 30 seconds.

#### Fabrication of dry electrodes with conductive desktop inkjet printing

The fabrication method is based on prior work, which used a desktop inkjet printer to print functional traces on various substrate materials^19,34^. Commercial tattoo decal paper (SUNNYSCOPA, printable temporary tattoo paper for laser printer) was used as the substrate material. A layer with electrodes and connecting traces was printed using silver nanoparticle ink (Sicrys™ I40DM-106) and heat cured. An additional three layers of PEDOT:PSS (Orgacon™ IJ-1005, 739316) conductive polymer using the same design were printed to enhance the mechanical robustness of the brittle metallic traces. Routing traces, but not electrodes, were then insulated by printing five layers of PVP (Polyvinylphenol, Mw = 11,000 g/mol) on top. The layers were thermally cured. A sheet of skin adhesive film (SUNNYSCOPA) was laser cut to leave electrode locations uncovered and then bonded onto the printed tattoo sheet. The sandwich was then transferred onto the skin.

#### Hardware and interfacing

Custom hardware setups were implemented for recording EMG and EDA signals based on existing open-source hardware specifications. For EMG, our hardware setup is based on prior work, which presented solutions for recording high-quality EMG data^57–59^. The sEMG acquisition board consists of one differential amplifier (INA331IDGKT, Texas Instruments) and two zero-drift amplifiers (OPA333, Texas Instruments) and can measure the EMG signal of one muscle through three electrodes (two measurements and one reference). The acquisition board converts the analog differential signal (the EMG bio-potentials generated by muscles) attached to its inputs through a disposable surface electrodes connector into a single stream of data as output. The output signal is analog and has to be discretized for digital processing. The signal is passed through an instrumentation amplifier (Gain = 10) followed by a high-pass filter with a cut-off frequency of 0.2Hz. Finally, an operational amplifier with a regulated gain (in the range [5.76, 101]) was used for producing a filtered amplified signal. The electrodes (measurement and reference) are connected to the board through an audio jack (aux cable). For supporting multiple muscles, multiple sEMG boards were connected with one common reference electrode. For EDA signal acquisition, an open-source hardware platform was used^56^. The hardware units were externally grounded.

## Supplementary information

Supplementary Information

Description of Additional Supplementary Files

Supplementary Data 1

Supplementary Movie 1

Supplementary Movie 2

Supplementary Movie 3

## Data Availability

All data generated or analyzed during this study are included in the published article and its Supplementary Information. Additional raw data is hosted on the OSF (Open Science Framework) platform and can be found at the following link: https://osf.io/zerqx/?view_only=cf2a719779134c59a0bf35a2e642c2f2.

## Code availability

Custom code used in the current study is available from the corresponding author on request.
