# Supplementary material for: Computational design and optimization of electro-physiological sensors
Source: Nat Commun. 2021 Nov 3;12:6351. doi: 10.1038/s41467-021-26442-1 (PMC8566494; doi:10.1038/s41467-021-26442-1)
Supplement: Supplementary file 1 — Supplementary Information [file 41467_2021_26442_MOESM1_ESM.pdf]

Supplementary Information: Computational Design and Optimization of Electro-Physiological Sensors

Aditya Shekhar Nittala[1], Andreas Karrenbauer[2], Arshad Khan[1,3], Tobias Kraus[3,*], and Jürgen Steimle[1,*]

[1]Human Computer Interaction Lab, Saarland University, Saarland Informatics Campus, Saarbrücken, 66123, Germany
[2]Max Planck Institute for Informatics, Saarland Informatics Campus, Saarbrücken, 66123, Germany
[3]INM - Leibniz Institute for New Materials, Saarbrücken, 66123, Germany
[*]steimle@cs.uni-saarland.de, tobias.kraus@leibniz-inm.de

Modelling and Identification of Muscle Lines Given the four measurements of the forearm, the entire space of the anterior side of the forearm can be modelled as a trapezoid. From this, the muscle lines for Brachioradialis (BR), Flexor Carpi Radialis (FCR), Palmaris Longus (PL), Pronator Quadratus (PQ) and Flexor Carpi Ulnaris (FCR) can be reconstructed. The FCR muscle line can be identified as the line from the medial epicondyle to the radial styloid process[1] (which forms the diagonal of the trapezoid as shown in Figure 1). The BR muscle line can be identified as the line from the styloid process to a midpoint on the line between the lateral and medial epicondyles[1] (which is represented by the side d of the trapezoid a shown in Figure 1). The PL muscle line is the line between the medial epicondyle and the distal end of the flexor retinaculum[1] (which is represented by the line joining one corner of trapezoid a and the mid-point of b as shown in Figure 1). The PQ muscle line is identified as the horizontal muscle line situated at 2.5 cm from the wrist (Sulcus Distal Carpi)[2]. The muscle line runs parallel to the measurements a and b as shown in Figure 1. The FCU muscle line can be identified as muscle line from the medial epicondyle and ends near the other end of the styloid process[3] (which is represented by the side c of the trapezoid as shown in Figure 1).

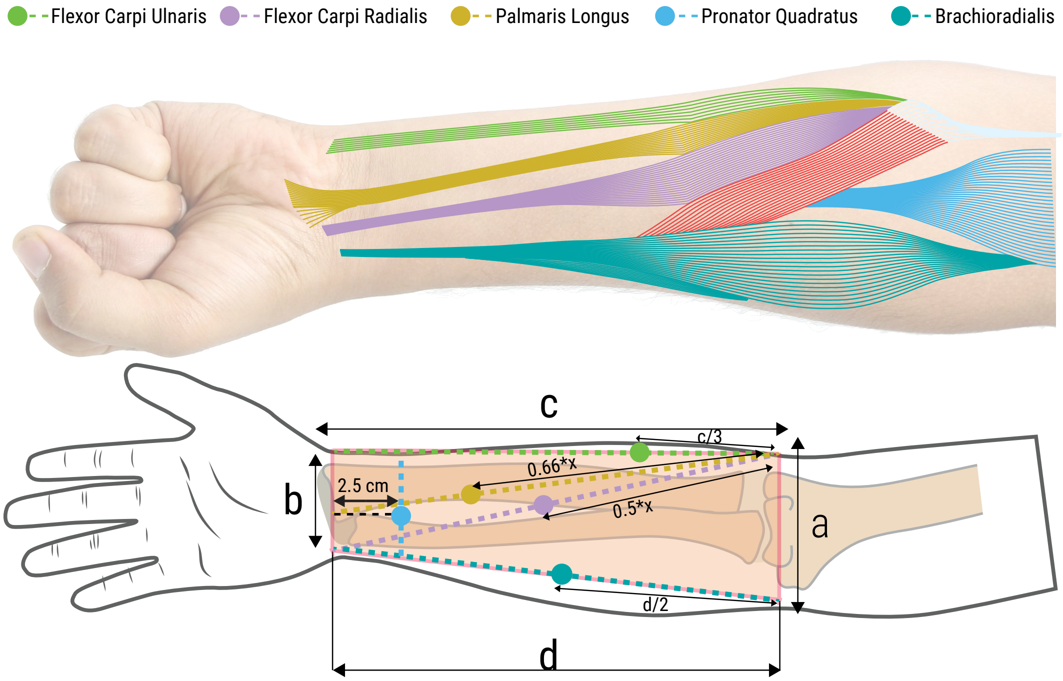

Supplementary Figure 1. Key points generated for each of the muscles on the anterior side of the forearm. The electrodes need to be placed symmetrically along this key point. ("x" denotes the length of the muscle line). The four measurements a , b, c, d for the forearm form the basis for the calculation of the muscle keypoints on the anterior side of the forearm.

Estimating the Count of Sweat Glands For two given circular electrodes, the area covered by and between the electrodes can be calculated as shown in Figure 2. Hence, if D_s is the density of sweat glands at a specific location, the number of sweat glands covered by the electrodes is given by: $N_s = (\pi r^2 + (d \times 2r)) \times D_s$.

ECG Keypoints on the Forearm To the best of our knowledge there exists no continuous models which predict the strength of ECG signals based on the spatial configuration of the measuring electrodes on the forearm. Therefore, we adopted a discrete model based on prior work[4]. ECG measurements were taken for a set of discrete locations on the forearm. These locations were chosen from prior work[4]. Figure 3 shows the key point locations incorporated into the model and the SNRs measured with a portable ECG device for combinations of key points. The SNR measurements were highest at the upper end of the forearm since the electrodes are closer to the heart. The signals drop drastically as the electrodes are placed farther down on the forearm.

Comparison of Optimizer Results with Conventional Designs Figure 4(a) shows the sampling of the forearm space used for generating optimized solutions. Figure 4(b) shows the scatter plot of all solutions generated for the uni-modal case, plotted against their respective area. It can be observed that the smallest possible solution can be easily identified by the solution which is present on the extreme left. This specific solution was generated when the window height was 3cm. Below this threshold, no valid solution were found because there was not sufficient space for the optimizer to fit in all the electrodes. Conversely, for identifying the solution with the best quality while having a small size, the search space was decreased in increments, until the predicted signal quality dropped below 90%. The QUALITY OPTIMIZED solution can then be easily recognized as the solution with

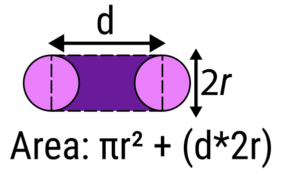

Supplementary Figure 2. Total area covered between and under two EDA electrodes.

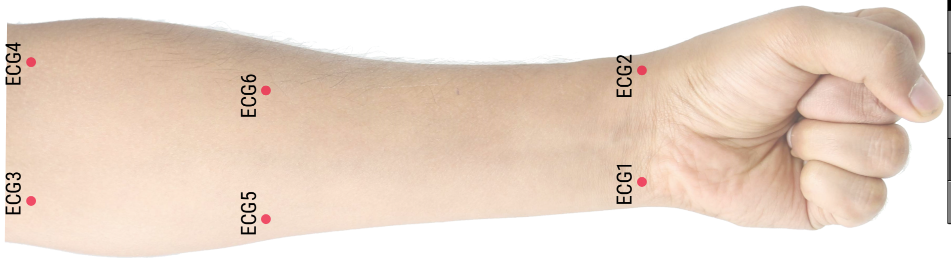

ECG Pair	SNR
(ECG1, ECG2)	4.36
(ECG3, ECG4)	17.44
(ECG5, ECG6)	8.27
(ECG1, ECG6)	5.16
(ECG4, ECG5)	9.39

Supplementary Figure 3. Key points for ECG electrode placement on the forearm and empirically measured signal quality for combinations of key points.

smallest area out of all solutions that have >=95% predicted quality, or <=0.05 optimizer score as shown in Figure 4(b).

EMG Measurements Figures 5 and 6 show the average rectified EMG signals for the BASELINE, QUALITY OPTIMIZED, AREA OPTIMIZED and EXPERT GENERATED sensor designs for the uni-modal and multi-modal configuration respectively. Each subplot shows the data from the five movement trials for each condition and muscle (please refer to EMG Data Collection in the Methods Section). It can be noticed that for all sensor designs, the muscle activation can be clearly recognized from the peaks. The quality of the signal was measured by calculating the ARV value over the window where the signal is present. One of the key observations here is that, for cases in which the Optimizer predicts the worst quality (optimizer score of 1), there is still a weak signal (see Figure 5, Expert-Generated signal for FCR). This is because the optimizer has been modelled with hard constraints (e.g. off set to muscle line of 1cm results in zero quality) to ensure that the signal quality of the resulting device is usually high.

Skin Conductance Measurements The skin conductance levels for each of the designs is shown in Figure 7. The results show that the information is still retained, although the skin conductance levels are lower when compared to the reference level, owing to the lower density of sweat glands covered by the sensor design. The average skin conductance level was 3.67μS, 3.63μS, 3.58μS, 2.47μS for the BASELINE SOLUTION, QUALITY OPTIMIZED, AREA OPTIMIZED, and EXPERT GENERATED solutions respectively. The EXPERT GENERATED solution had lower skin conductance level owing to the fact that the inter-electrode distance between the electrodes was ~4 cm while that other solutions had an inter-electrode distance of ~6 cm. The smaller inter-electrode distance resulted in a drop in the skin conductance levels because the number of sweat glands covered were lower when compared to other solutions. For all the sensor solutions, the reference skin conductance level was also measured by placing a commercial EDA sensor consisting of dry metallic electrodes (Seeed Studio Groove[5]) on the fingers. One electrode was placed on the index finger while the other electrode was placed on the middle finger.

ECG Measurements Figure 8 shows the raw ECG signals for each of the four solutions. It can be clearly seen that the BASELINE SOLUTION and QUALITY OPTIMIZED solutions have similar quality in the ECG signals. Though the SNR levels of the AREA OPTIMIZED and EXPERT OPTIMIZED are lower when compared to BASELINE SOLUTION, the signal can still be used for detecting Heart Rate Variability (HRV) which can be beneficial for various scenarios such as in applications in Virtual Reality and Human-Machine Interaction.

Signal Quality of Dry Electrodes We briefly summarize previous work[6] where we measured and compared the performance of commercial wet-gel electrodes (Kendall^{TM} Covidien H124SG) with dry electrodes that were fabricated using the same conductive ink-jet printing method and temporary tattoo paper as used in this work, for the readers' convenience. The experiment was conducted with multiple subjects (8 participants, 3 female, average: 28.5 years). The results from this experiment indicate that the dry electrodes can capture EMG signals with high quality, with a decrease in SNR of ~5% when compared to commercial gel electrodes. For the EDA signals, the dry electrodes achieved a high Pearson correlation coefficient (mean: 0.95, SD: 0.01). For ECG signals, the dry electrodes had a decrease of ~15% in the SNR levels when compared to the gel

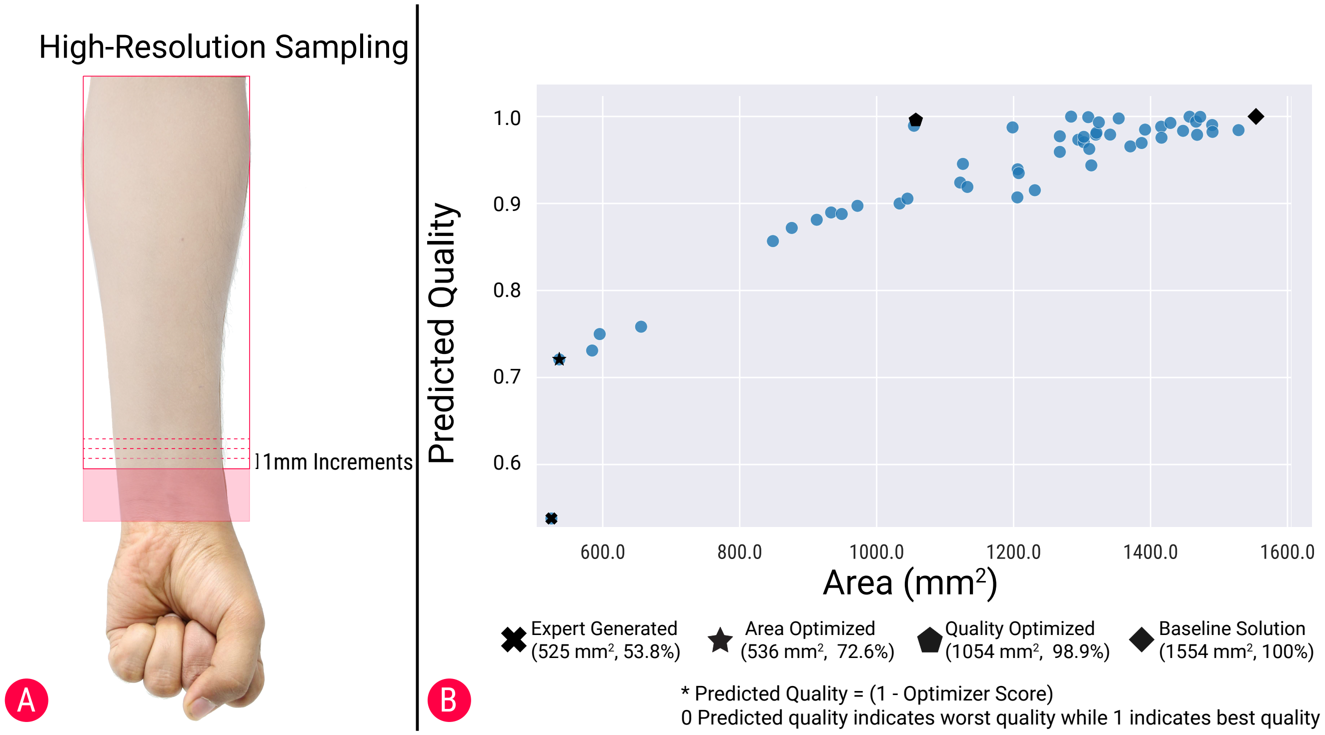

Supplementary Figure 4. (a) High resolution sampling of the anterior side of the forearm in 1 mm increments starting near the wrist. (b) Scatter plot showing the entire spectrum of solutions generated by varying the search space on the forearm. The solutions have been plotted with respect to their size.

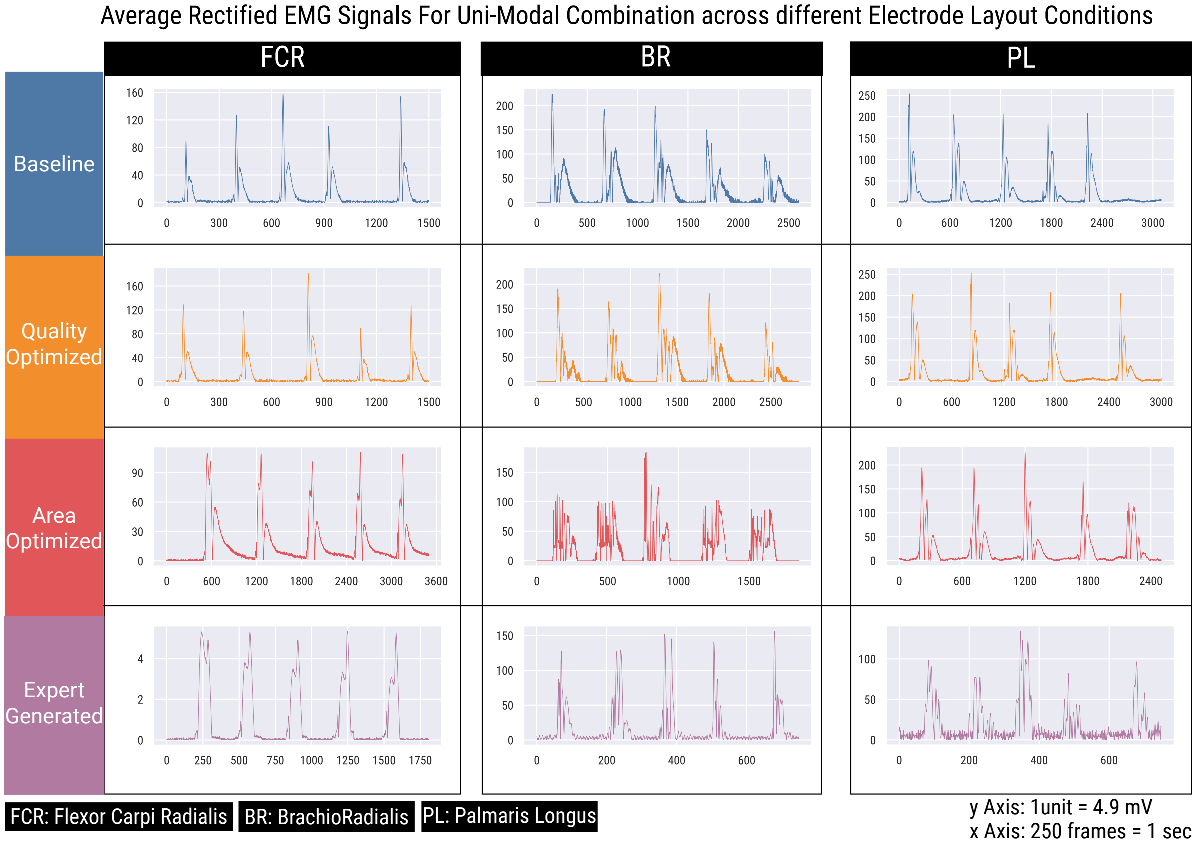

Supplementary Figure 5. Average rectified EMG signals for the uni-modal combination consisting of three muscles. For each of the sensor design condition and the muscle, the predicted vs. the measured qualities have been labelled.

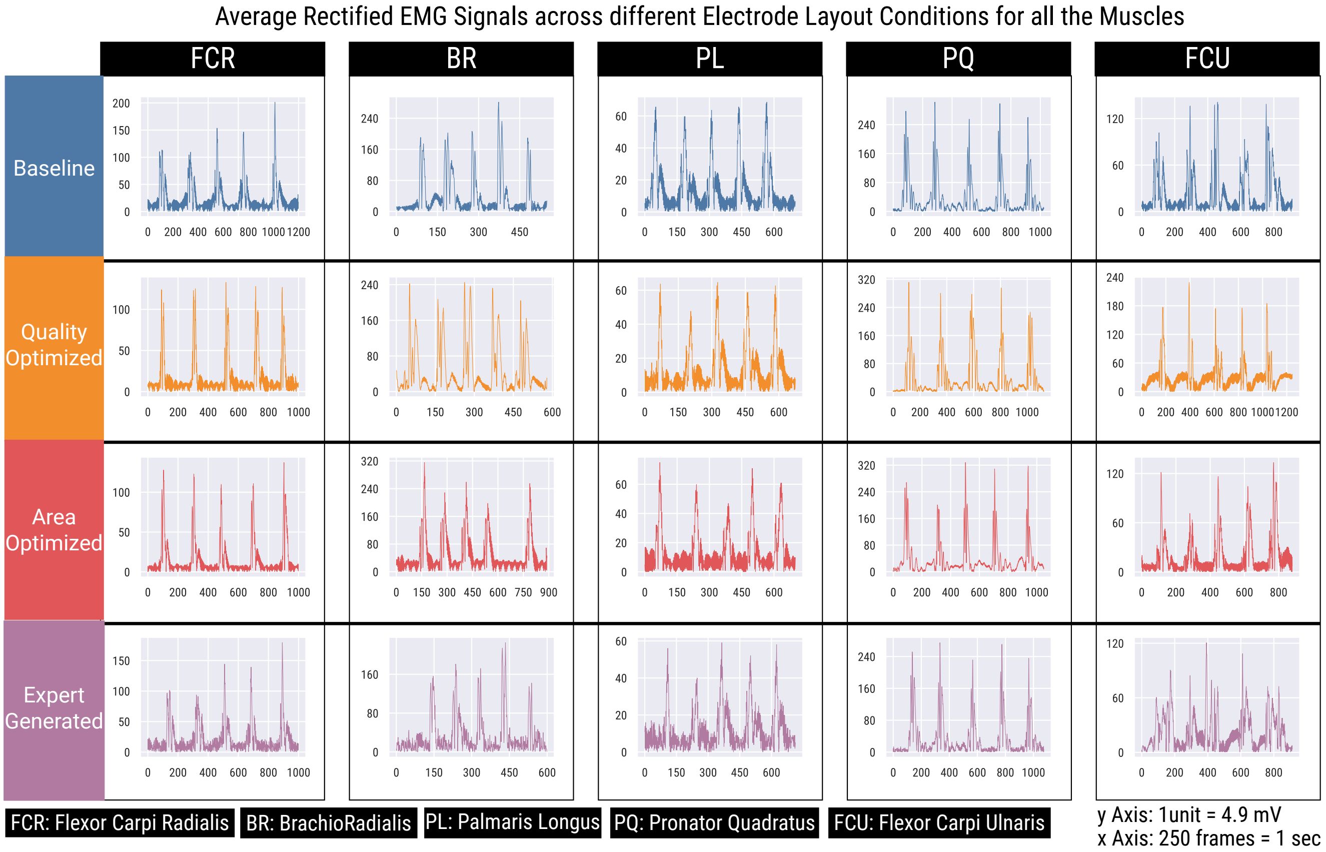

Supplementary Figure 6. Average Rectified EMG signals for each of the muscles for all sensor design solutions in the multi-modal case.

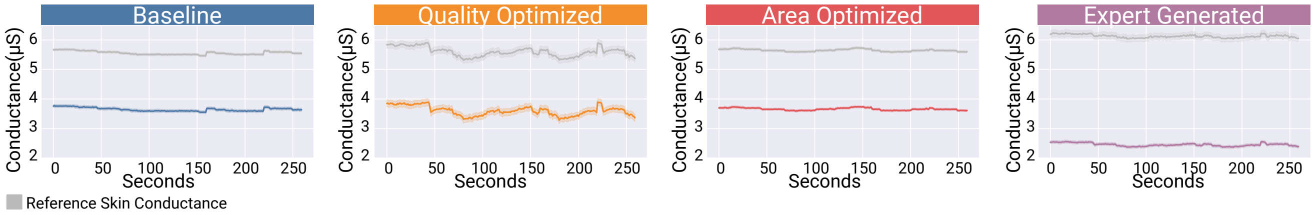

Supplementary Figure 7. Raw signals of the EDA measurements for a participant for all the sensor design solutions. During each measurement, a reference measurement of skin conductance was taken by placing the electrodes on the fingertips.

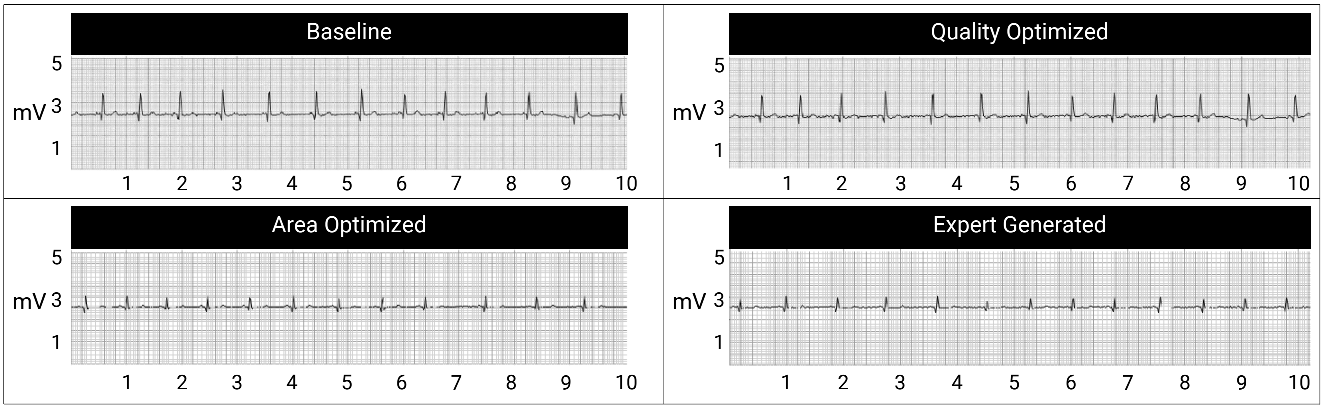

Supplementary Figure 8. Raw signals of the ECG measurements for a participant for all four solutions.

electrodes and can produce meaningful ECG recordings. These results demonstrate that the dry electrodes fabricated through ink-jet printing have the capability to capture bio-signals with sufficiently high levels of quality required for non-medical applications[7–9]. Refer to the original publication[6] for additional technical details.

Accuracy of Optimizer Predictions for Dry Electrodes We performed an additional experiment to understand how the optimizer's prediction scales to dry electrodes. In contrast to prior work[6] which placed electrodes at the most ideal locations, this experiment evaluates the signal quality of the electrodes when they are placed at non-ideal locations specified by the optimizer.

Method: The multi-modal combination was chosen for this experiment along with the Area Optimized solution generated by the optimizer. This demanding case covered all supported modalities and muscles in a compact form factor. For comparison, signals were also captured with gel electrodes (Kendall™ Covidien H135SG, Sensor Area: 50mm^2 for EMG and ECG, Kendall™ Covidien H124SG, Sensor Area: 80mm^2 for EDA) using the same electrode layout. Overall, this experiment had the following four conditions:

- Gel Electrodes - Baseline: The gel electrodes are placed at the most ideal locations on the forearm as specified by the Baseline electrode layout (see main article).
- Gel Electrodes - Area Optimized: The gel electrodes are placed at the optimized locations on the forearm as specified by the Area Optimized electrode layout.
- Dry Electrodes - Baseline: The dry electrodes are placed at the most ideal locations on the forearm as specified by the Baseline solution.
- Dry Electrodes - Area Optimized: The dry electrodes are placed at the optimized locations on the forearm as specified by the Area Optimized solution.

For each of the conditions, the signals for EMG (for all five muscles), EDA and ECG modalities were captured. For the dry electrodes, electrodes of circular shape were fabricated on a temporary tattoo paper substrate using the conductive ink-jet printing technique described in the Methods section. The dry electrodes had a diameter of 50 mm^2 for EMG and ECG modalities and 80 mm^2 for EDA. The same data collection method was used as described in the Methods section. One of the subjects who participated in the experimental validation of the optimizer was chosen for this experiment. To mitigate order effects, each modality was chosen at random and the order of presentation for dry and gel electrodes was chosen randomly.

Results: Figure 9 shows the comparison of the values that were predicted by the optimizer with the experimentally measured values, both for commercial gel electrodes and dry electrodes. The experimentally measured values were normalized with respect to the quality of the signal obtained in the Baseline condition of the respective type of electrode. It can be noticed that the quantitative agreement between the optimizer prediction and the experimentally measured signal quality is at similar levels for both types of electrodes, for all three modalities. This finding suggests that the optimization approach can be generalized to different types of electrodes provided the electrodes can capture the biosignals with high quality.

Figure 11 (A) shows the absolute average rectified values of EMG signals for five muscles, captured using the Baseline and Area Optimized layouts with gel electrodes. Five trials were captured for each muscle in each condition. For comparison, Figure 11 (B) shows the values obtained using dry electrodes. The mean ARV of signals captured with gel electrodes (measured for both Baseline and Area Optimized conditions) was 1.05 V. The mean ARV of signals captured with dry electrodes (measured for both Baseline and Area Optimized solutions) was 0.91 V. This reduction in ARV levels is in-line with findings reported in previous work [6], which showed an average drop of ~9%. A key observation here is that while there is a drop in the signal quality for the dry electrodes in comparison to gel electrodes, the predicted and measured accuracies still are close for dry electrodes since we normalize with respect to the Baseline solution of dry electrodes.A similar trend is also noticeable for the skin conductance measurements. It is expected that there are large variations over the course of a day. The skin conductance measurements were 9.85μS, and 9.56μS for the Gel-Baseline and Gel - Area Optimized conditions. The skin conductance measurements for the Dry Electrodes - Baseline and Dry Electrodes - Area Optimized solution were 12.76μS and 12.09μS respectively. It is interesting to note that while there is change in the skin conductance levels for the Baseline solution for the gel and dry electrodes respectively, the optimized solutions still obtains very high levels of skin conductance when compared to their respective Baseline solutions. A similar trend is also observed for the ECG signals. The SNR levels for the Gel Electrodes- Area Optimized and Dry Electrodes - Area Optimized are 3.35 (sd: 0.30) and 2.67(sd:0.71) respectively. The ECG signals for each of the experimental conditions is shown in Figure 13.

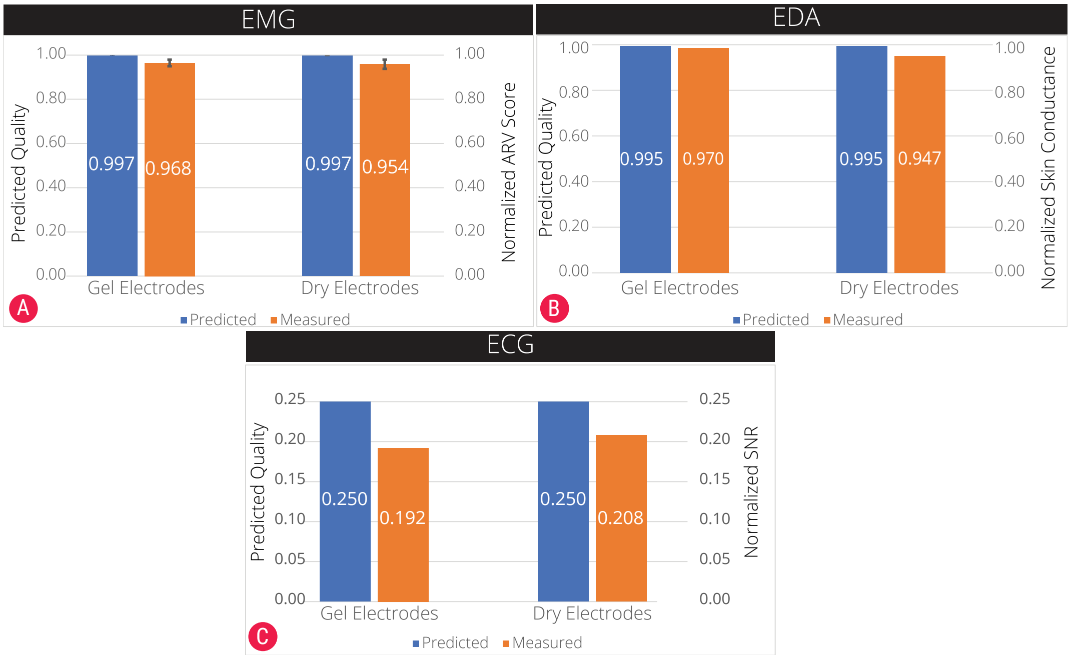

Supplementary Figure 9. Accuracy of optimizer predictions for gel electrodes and dry electrodes for the Area Optimized solution. (a) Comparison of EMG signal quality predicted by the optimizer and normalized experimental measurement, for gel electrodes and dry electrodes. (b) Comparison of EMG signal quality predicted by the optimizer and normalized experimental measurement, for gel electrodes and dry electrodes (c) Comparison of EMG signal quality predicted by the optimizer and normalized experimental measurement, for gel electrodes and dry electrodes.

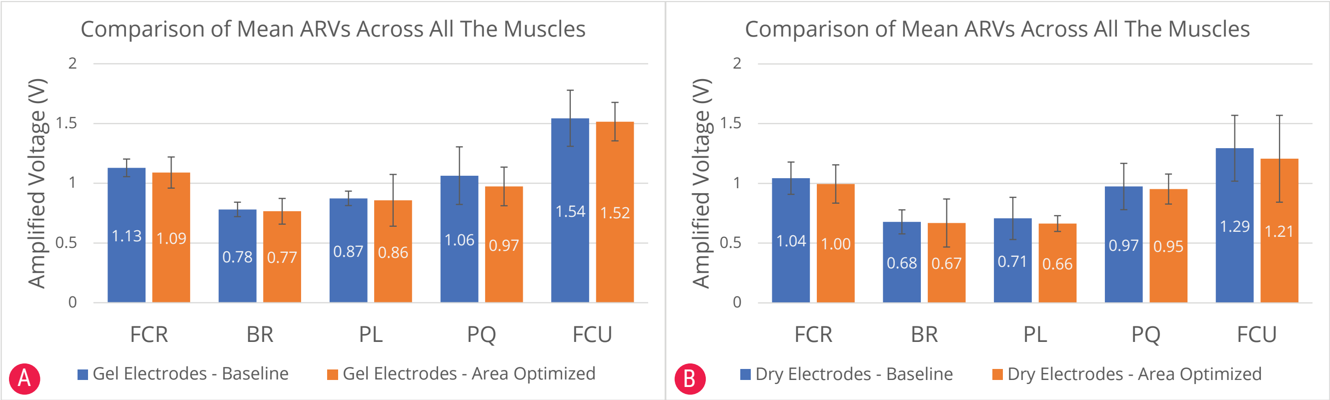

Supplementary Figure 10. Magnitude of the raw ARV signals of EMG measurements for five muscles (a) Pair-wise comparison of the ARVs for the gel electrodes for Baseline and Area Optimized placement conditions (b) Pair-wise comparison of the ARVs for the dry electrodes for Baseline and Area Optimized conditions.

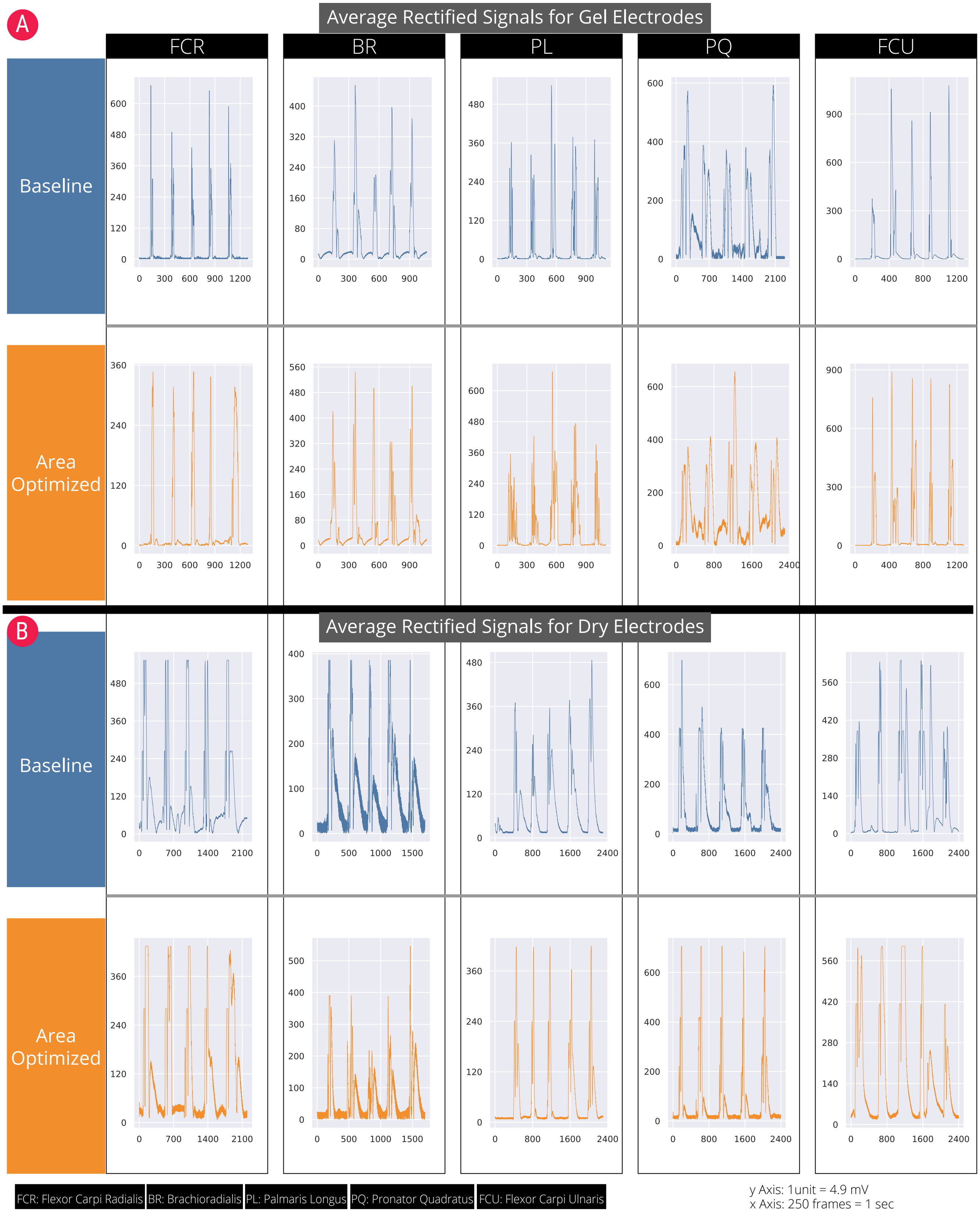

Supplementary Figure 11. (a) Raw ARV signals of EMG measurements for each of the five muscles. (a) Raw ARV signals for Gel Electrodes -Baseline and Gel Electrodes - Area Optimized conditions. (b) Raw ARV signals for Same signals obtained with dry electrodes.

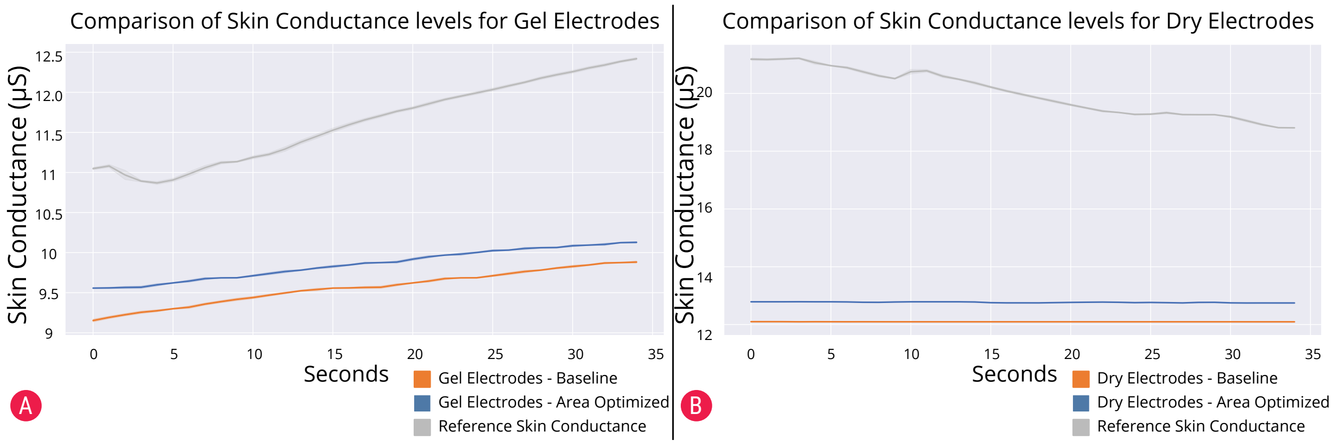

Supplementary Figure 12. Raw signals of EDA measurements. (a) Skin conductance levels for the gel electrode in BASELINE and AREA OPTIMIZED conditions. (b) Skin conductance levels in the BASELINE and AREA OPTIMIZED CONDITIONS FOR THE DRY ELECTRODES.

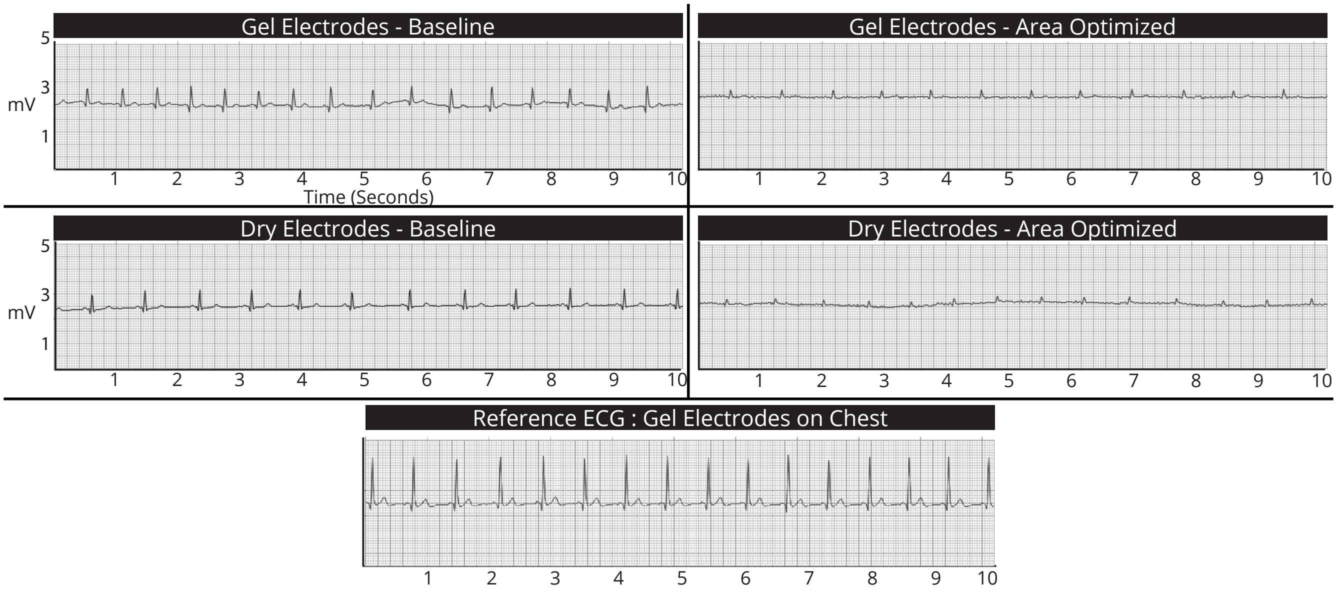

Supplementary Figure 13. Raw signals of the ECG measurements showing the comparison of signals with Gel Electrodes and Dry Electrodes on the forearm.

References

1. Barbero, M., Merletti, R. & Rainoldi, A. *Atlas of muscle innervation zones: understanding surface electromyography and its applications* (Springer Science & Business Media, 2012).
2. Zipp, P. Recommendations for the standardization of lead positions in surface electromyography. *Eur. journal applied physiology occupational physiology* **50**, 41–54, DOI: 10.1007/BF00952243 (1982).
3. Delagi, E. F. *Anatomical guide for the electromyographer: the limbs and trunk* (Charles C. Thomas, 2011).
4. Escalona, O. J. *et al.* Wrist and arm body surface bipolar ecg leads signal and sensor study for long-term rhythm monitoring. In *2017 Computing in Cardiology (CinC)*, 1–4, DOI: 10.22489/CinC.2017.071-458 (IEEE, 2017).
5. Seeed Studio Groove gsr. http://wiki.seeedstudio.com/Grove-GSR_Sensor/. Accessed: 2021-07-23.
6. Nittala, A. S., Khan, A., Kruttwig, K., Kraus, T. & Steimle, J. PhysioSkin: Rapid fabrication of skin-conformal physiological interfaces. In *Proceedings of the SIGCHI Conference on Human Factors in Computing Systems (CHI '20)*, DOI: 10.1145/3313831.3376366 (ACM, 2020).
7. Ferrari, L. M. *et al.* Ultraconformable temporary tattoo electrodes for electrophysiology. *Adv. Sci.* **5**, 1700771, DOI: 10.1002/advs.201700771 (2018).
8. Bonato, P., D'Alessio, T. & Knaflitz, M. A statistical method for the measurement of muscle activation intervals from surface myoelectric signal during gait. *IEEE Transactions on biomedical engineering* **45**, 287–299 (1998).
9. Haddad, P. A., Servati, A., Soltanian, S., Ko, F. & Servati, P. Effects of flexible dry electrode design on electrodermal activity stimulus response detection. *IEEE Transactions on Biomed. Eng.* **64**, 2979–2987, DOI: 10.1109/TBME.2017.2754220 (2017).
