# Supplementary material for: Computational design and optimization of electro-physiological sensors
Source: Nat Commun. 2021 Nov 3;12:6351. doi: 10.1038/s41467-021-26442-1 (PMC8566494; doi:10.1038/s41467-021-26442-1)
Supplement: Supplementary file 2 — Description of Additional Supplementary Files [file 41467_2021_26442_MOESM2_ESM.pdf]

Description of Additional Supplementary Files

File name: Supplementary Data 1

Description: The file contains the experimental data collected during our study. The Excel sheet also contains the plots that have been presented in the main article.

File name: Supplementary Movie 1

Description: Supplementary Movie 1 demonstrates the interactive optimizer that has been implemented through a web-based software tool. It shows the workflow for using the tool and generating optimized electrode layouts.

File name: Supplementary Movie 2

Description: Supplementary Movie 2 demonstrates the two applications that are described in the main manuscript.

File name: Supplementary Movie 3

Description: Supplementary Movie 3 demonstrates two additional optimization schemes (Lower Bound Optimization and Lower Bound Optimization with weights) that have been detailed in the main manuscript.
